# Hepatocyte TRAF3 promotes liver steatosis and systemic insulin resistance through targeting TAK1-dependent signalling

**DOI:** 10.1038/ncomms10592

**Published:** 2016-02-17

**Authors:** Pi-Xiao Wang, Xiao-Jing Zhang, Pengcheng Luo, Xi Jiang, Peng Zhang, Junhong Guo, Guang-Nian Zhao, Xueyong Zhu, Yan Zhang, Sijun Yang, Hongliang Li

**Affiliations:** 1 Department of Cardiology, Renmin Hospital of Wuhan University, Wuhan 430060, China; 2 Animal Experiment Center/Animal Biosafety Level-III Laboratory, Wuhan University, Wuhan 430060, China; 3 State Key Laboratory of Quality Research in Chinese Medicine, Institute of Chinese Medical Sciences, University of Macau, Macao 999078, China; 4 Department of Nephrology, Renmin Hospital of Wuhan University, Wuhan 430060, China; 5 Huangshi Central Hospital, Hubei Polytechnic University, Huangshi 435000, China

## Abstract

Non-alcoholic fatty liver disease (NAFLD) is characterized by hepatic steatosis, insulin resistance and a systemic pro-inflammatory response. Here we show that tumour necrosis factor receptor-associated factor 3 (TRAF3) is upregulated in mouse and human livers with hepatic steatosis. After 24 weeks on a high-fat diet (HFD), obesity, insulin resistance, hepatic steatosis and inflammatory responses are significantly ameliorated in liver-specific TRAF3-knockout mice, but exacerbated in transgenic mice overexpressing TRAF3 in hepatocytes. The detrimental effects of TRAF3 on hepatic steatosis and related pathologies are confirmed in *ob/ob* mice. We further show that in response to HFD, hepatocyte TRAF3 binds to TGF-β-activated kinase 1 (TAK1) to induce TAK1 ubiquitination and subsequent autophosphorylation, thereby enhancing the activation of downstream IKKβ–NF-κB and MKK–JNK–IRS1^307^ signalling cascades, while disrupting AKT–GSK3β/FOXO1 signalling. The TRAF3–TAK1 interaction and TAK1 ubiquitination are indispensable for TRAF3-regulated hepatic steatosis. In conclusion, hepatocyte TRAF3 promotes HFD-induced or genetic hepatic steatosis in a TAK1-dependent manner.

## Abstract (web summary)

TRAF family proteins regulate immune signalling cascades. Here, the authors show that TRAF3 is upregulated in the liver in non-alcoholic fatty liver disease, promoting insulin resistance, inflammation and hepatic steatosis via its interaction with the kinase TAK1.

## Graphical abstract

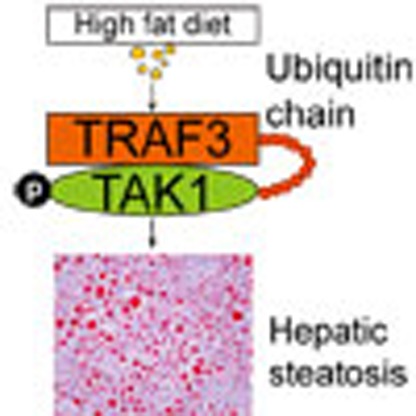

As a primary metabolic organ in the human body, the liver plays a key role in the regulation of lipid metabolism and is sensitive to energy intake^1^,^2^. Hepatic steatosis is an early pathological condition of the liver and can lead to steatohepatitis, cirrhosis, liver failure and severe cardiovascular diseases^3^,^4^. Accumulating evidence indicates that hepatic steatosis is a low-grade inflammatory response and commonly occurs in individuals with insulin resistance and obesity^5^. In the liver, the impaired insulin signalling pathway, which involves the insulin receptor substrate (IRS) proteins and AKT cascade, results in insulin insensitivity and glucose intolerance. This impairment in insulin signalling, together with inflammatory response, promotes hepatic lipid synthesis and steatosis, which in turn contribute to chronic hepatic inflammation and insulin resistance^6^,^7^. Thus, insulin resistance, inflammatory response and hepatic steatosis are interconnected pathological events that are often observed in obese individuals. Although extensive research has been conducted in this area, the complex interlinked molecular events and related cellular behaviours that occur during the initiation and progression of hepatic steatosis are not fully understood.

The tumour necrosis factor receptor (TNFR)-associated factor (TRAF) family consists of seven members (TRAF1–TRAF7) that can function as signalling adaptors in various pathophysiologic processes^8^,^9^. Among TRAF members, TRAF3 was first identified as a protein interacting with CD40 cytoplasmic domain and is ubiquitously expressed^10^,^11^. In mice, TRAF3-knockout (KO) is postnatal lethal, indicating a vital role of TRAF3 for development and growth^12^. TRAF3 can also act as an adaptor/transducer coupling receptors with their downstream factors and thus keeping intricate effects in regulating cell cycle and various cellular responses^13^,^14^,^15^,^16^. Nuclear factor-κB (NF-κB) and mitogen-activated protein kinase (MAPK) signalling are two of the most investigated downstream pathways regulated by TRAF3, and most observations supported a negative regulation of TRAF3 on non-canonical NF-κB cascade and JNK/P38 MAPK subunits^17^,^18^,^19^. In the recent years, TRAF3-controlled canonical NF-κB signalling was also been reported by Bista *et al*.^20^and Perez de Diego *et al*.^21^ however, with opposite results. Our recent studies demonstrated that neuronal TRAF3 promotes JNK and classic NF-κB pathways through a TAK1-dependent mechanism during stroke^22^. The increased NF-κB and JNK activations by TRAF3 were also found in hepatocytes on hepatic ischaemia/reperfusion injury^23^. These previous studies collectively indicated that TRAF3-regulated molecular events and cellular responses are largely cell type- and stress-dependent. Considering the close implication of NF-κB/JNK in insulin function and inflammatory response that intimately associated with fatty acid metabolism in hepatocytes^24^, we hypothesize that TRAF3 might be involved in the pathogenesis of hepatic steatosis. In fact, Chen *et al*.^25^ recently reported that TRAF3 in myeloid cells exacerbates insulin resistance and liver lipid droplet. However, whether TRAF3 in hepatocytes can mediate hepatic steatosis remains unknown.

Given that hepatocytes are the primary cell type that is damaged during hepatic steatosis, in our present study, hepatocyte-specific TRAF3-KO and TRAF3-transgenic mice are generated and subjected to an high-fat diet (HFD) to evaluate the effects of TRAF3 on hepatic steatosis and related metabolic disorder. We identify positive regulatory role of hepatocyte TRAF3 in hepatic steatosis, insulin resistance and inflammatory response.

## Results

### TRAF3 is upregulated in livers with hepatic steatosis

**Figure 1:**
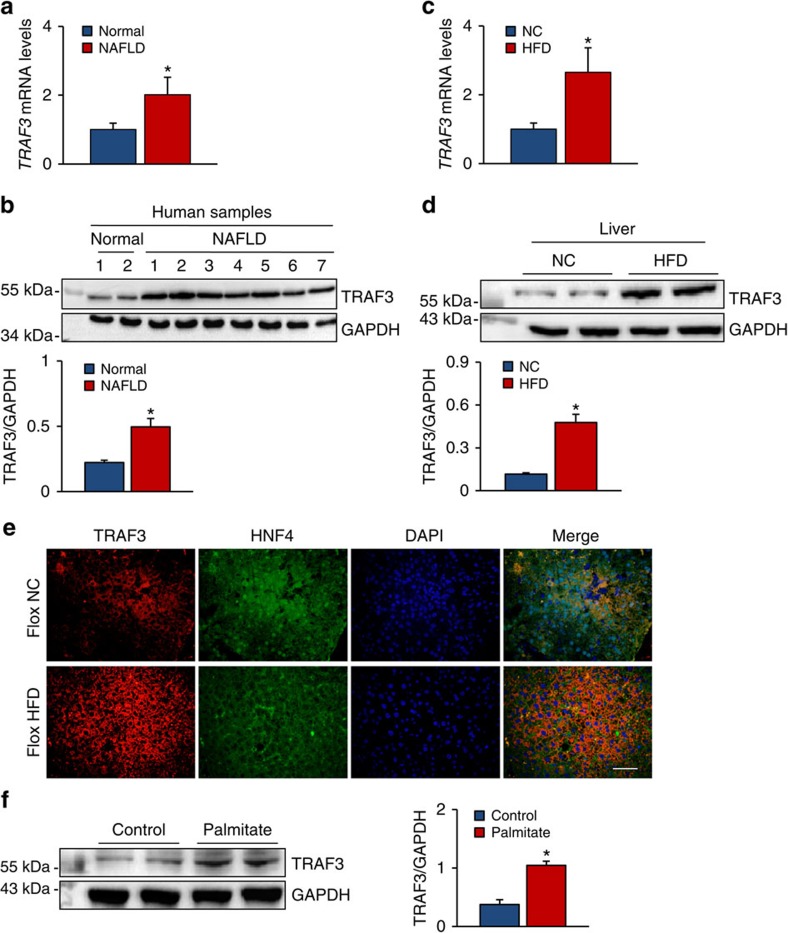
TRAF3 expression is upregulated in livers with hepatic steatosis. (**a**,**b**) The mRNA (**a**) and protein (**b**) levels of TRAF3 in liver biopsy samples from normal donors and patients with NAFLD. TRAF3 expression was normalized to that of GAPDH (*n*=4 for normal and 7 for NAFLD samples, *P<0.05 versus normal donors). (**c**,**d**) *TRAF3* mRNA (**c**) and protein (**d**) expression in the liver of mice subjected to an HFD for 24 weeks and in NC controls (*n*=6 samples per experimental group, *P<0.05 compared with the NC group). (**e**) Immunofluorescence images of staining with antibodies against HNF4 (green) and TRAF3 (red). Nuclei were labelled with DAPI (blue; *n*=8 samples per experimental group). Scale bar, 50 μm. (**f**) Primary hepatocytes were isolated and stimulated with palmitate (0.25 mM) for 24 h, and TRAF3 expression was examined by immunoblotting. GAPDH served as a loading control (*n*=6 samples per experimental group, *P<0.05 versus control). The data represent as the mean±s.d. Statistical analysis was carried out by Student's two-tailed *t*-test.

To investigate the involvement of TRAF3 in hepatic steatosis, *TRAF3* mRNA and protein expression levels in liver samples of non-alcoholic fatty liver disease (NAFLD) patients were examined. As shown in Fig. 1a,b, higher *TRAF3* mRNA and protein levels were observed in the livers of NAFLD patients than in healthy controls. Furthermore, TRAF3 expression levels were examined in the liver, muscle and fat of wild-type (WT) mice subjected to an HFD-induced hepatic steatosis. Consistent with the findings in human samples, both the mRNA and the protein expression of TRAF3 were significantly upregulated in the liver after 24 weeks of an HFD (Fig. 1c,d); however, increased TRAF3 expression was failed to be observed in the fat or muscle (Supplementary Fig. 1). Immunofluorescence experiments indicated that the increased expression of TRAF3 was primarily localized in the cytoplasm of hepatocytes, the nuclei of which were marked by an antibody specific for HNF4, a factor specifically expressed in hepatocytes^26^ (Fig. 1e). To directly visualize TRAF3 expression location on hepatocytes during lipid accumulation, palmitate was incubated with primary hepatocytes to mimic hepatic steatosis and insulin resistance *in vitro*^27^. Western blotting revealed that TRAF3 protein expression was markedly higher in palmitate-treated hepatocytes than in controls, after 24 h of incubation (Fig. 1f). The upregulated expression of TRAF3 in livers with hepatic steatosis suggests a potential involvement of TRAF3 in this pathological condition.

### TRAF3 deficiency ameliorates obesity and insulin resistance

**Figure 2:**
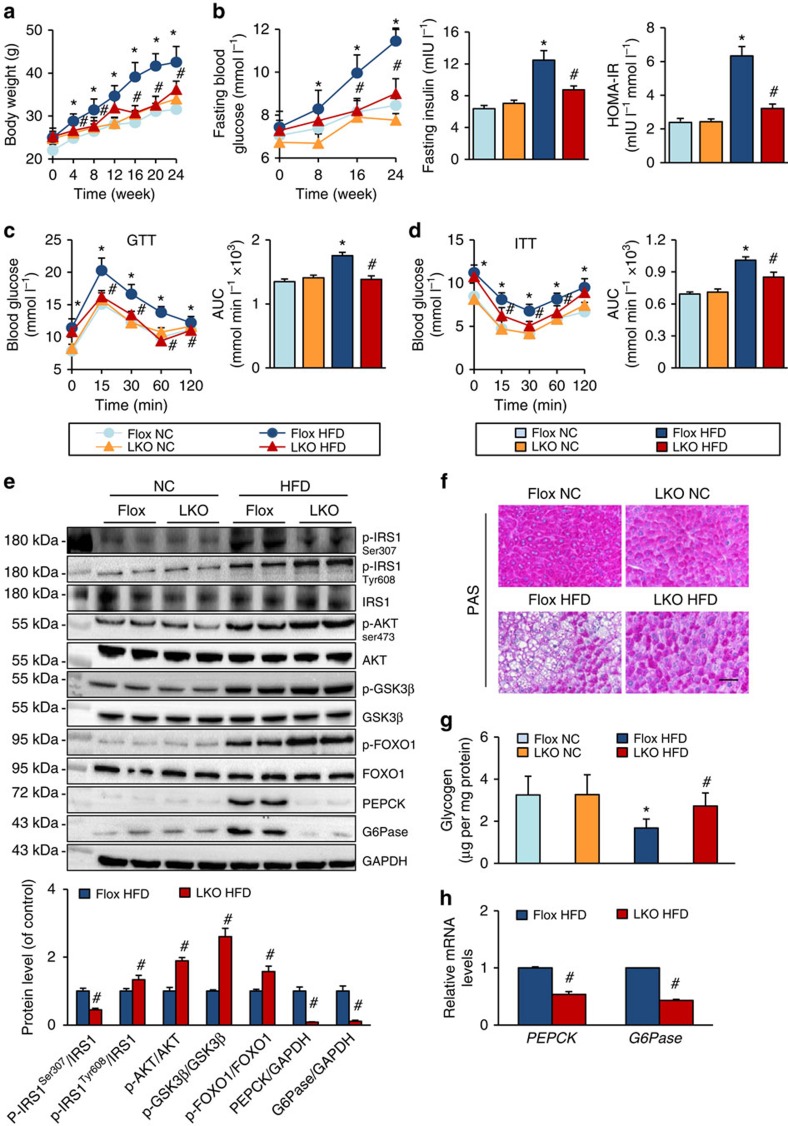
Liver-specific TRAF3 deficiency inhibits HFD-induced obesity and insulin resistance. (**a**) Changes in the body weight of TRAF3-LKO and TRAF3-flox mice treated with an HFD or NC for 24 weeks (*n*=16–25 for each group per time point). (**b**) FBG, FINS and HOMA-IR in TRAF3-LKO or TRAF3-flox mice treated with an HFD or NC for 24 weeks. FBG levels were measured every 4 weeks. FINS was measured at the end point of this experiment. HOMA-IR was calculated as HOMA-IR=(FBG (mmol l^−1^) × FINS (mIU l^−1^))/22.5 (*n*=8–12 for each group per time point). (**c**,**d**) Intraperitoneal glucose tolerance test (GTT) (1 g kg^−1^) (**c**) and intraperitoneal insulin tolerance test (ITT; 0.75 units per kg) (**d**) were performed on TRAF3-flox and TRAF3-LKO mice at the 22nd or 23rd week of food administered, respectively. The corresponding area under the curve (AUC) of blood glucose level was calculated (*n*=8–12 for each group). (**e**) Total and/or phosphorylated IRS1, AKT, GSK3β, FOXO1, PEPCK and G6Pase in liver samples of HFD-fed mice that were refed for 4 h after overnight fasting and NC mice under fasting state at the end of 24 weeks. GAPDH serves as the loading control (*n*=4 samples per experimental group). (**f**) Representative images of periodic acid–Schiff (PAS) staining on the liver sections of TRAF3-flox and TRAF3-LKO mice after a 24-week HFD or NC treatment. *n*=3–4 per group. Scale bar, 50 μm. (**g**) The glycogen content levels measured in the liver samples of mice in TRAF3-flox/NC, TRAF3-LKO/NC, TRAF3-flox/HFD and TRAF3-LKO/HFD groups. *n*=8–12 for each group. (**h**) The mRNA levels of *PEPCK* and *G6Pase* in the liver from mice in the indicated groups. *n*=4 for each group. For **a**–**h**, *P<0.05 compared with the corresponding TRAF3-flox/NC group; #*P*<0.05 compared with the corresponding TRAF3-flox/HFD group. All values are means±s.d. Significance determined by two-way analysis of variance with general linear model procedures using a univariate approach (**a**–**d**,**g**) and Student's two-tailed *t*-test (**e**,**h**).

Given that TRAF3 expression was exclusively increased in the livers of mice receiving an HFD, we generated mice with TRAF3 ablation in the liver (Supplementary Fig. 2a–c) and subjected them to an HFD. In response to HFD treatment, the body weight of TRAF3^flox/flox^ (TRAF3-flox) mice markedly increased during the period between 4 and 24 weeks compared with the normal chow (NC)-treated group, whereas the liver-specific TRAF3-KO (TRAF3-LKO) mice gained significantly less weight (Fig. 2a). However, there was no obvious difference in the energy intake between TRAF3-flox and TRAF3-LKO mice (Supplementary Fig. 2d), thereby excluding anorexia as the reason for the lower body weights of mice with hepatic TRAF3 deficiency. Adipose tissue accumulation in the viscera is always closely associated with and contributes to obesity^28^. Unsurprisingly, the visceral fat weight was increased in the TRAF3-flox mice by an HFD treatment for 24 weeks, but this increase was markedly suppressed in TRAF3-LKO mice without a significant change in the ratio of visceral fat weight to body weight (Supplementary Fig. 4a). In addition, the size of adipocytes was not significantly different between TRAF3-LKO and their floxed control littermates on HFD treatment (Supplementary Fig. 4b). This result suggests that blunted adipocyte proliferation, rather than enlargement, might contribute to the lower visceral fat weight in TRAF3-LKO mice than in the TRAF3-flox group.

Regarding the effect of hepatic TRAF3 on insulin resistance, we observed that fasting blood glucose (FBG) levels, fasting serum insulin (FINS) levels and homeostasis model assessment of the insulin resistance index (HOMA-IR) were significantly lower in TRAF3-LKO mice than in TRAF3-flox group after HFD administration (Fig. 2b). In addition, compared with the NC group, the area under the curve obtained from intraperitoneal glucose tolerance test and intraperitoneal insulin tolerance test assays was increased in HFD-treated TRAF3-flox mice, and these effects were markedly ameliorated by TRAF3-LKO (Fig. 2c,d).

Previous studies reported that the impairment of insulin sensitivity is largely mediated by insulin signalling, in particular the IRS–AKT–GSK3β cascade^29^. We therefore investigated the phosphorylated expression levels of IRS1, AKT and GSK3β in the liver of mice that were refed for 4 h after overnight fasting at 24 weeks of HFD administration, and observed that TRAF3 deficiency enhanced the activation of insulin signalling induced by refeeding, as shown by decreased phosphorylation of IRS1 at residue Ser307 and increased phosphorylated levels of IRS1 (Tyr608), AKT and GSK3β in the liver of TRAF3-LKO mice compared with the TRAF3-flox group (Fig. 2e). Moreover, periodic acid–Schiff staining and glycogen content analysis displayed that the amount of glycogen was greatly reduced in TRAF3-flox mice after HFD administration, but was largely preserved in the liver of the TRAF3-LKO group (Fig. 2f,g). In addition, the expression of the key gluconeogenesis enzymes PEPCK and G6Pase and their upstream FOXO1 that is regulated by Akt-mediated phosphorylation^30^,^31^ were examined. In line with the increased activation of insulin signalling, higher phosphorylation of FOXO1 and reduced expression of PEPCK and G6Pase were found in the liver of TRAF3-LKO mice than in TRAF3-flox mice after HFD administration (Fig. 2e,h). Collectively, the observations in TRAF3-LKO mice suggest that HFD-induced obesity, insulin resistance and glucose metabolic disturbance are inhibited by a liver-specific deficiency of TRAF3.

### TRAF3 overexpression exacerbates obesity and insulin resistance

**Figure 3:**
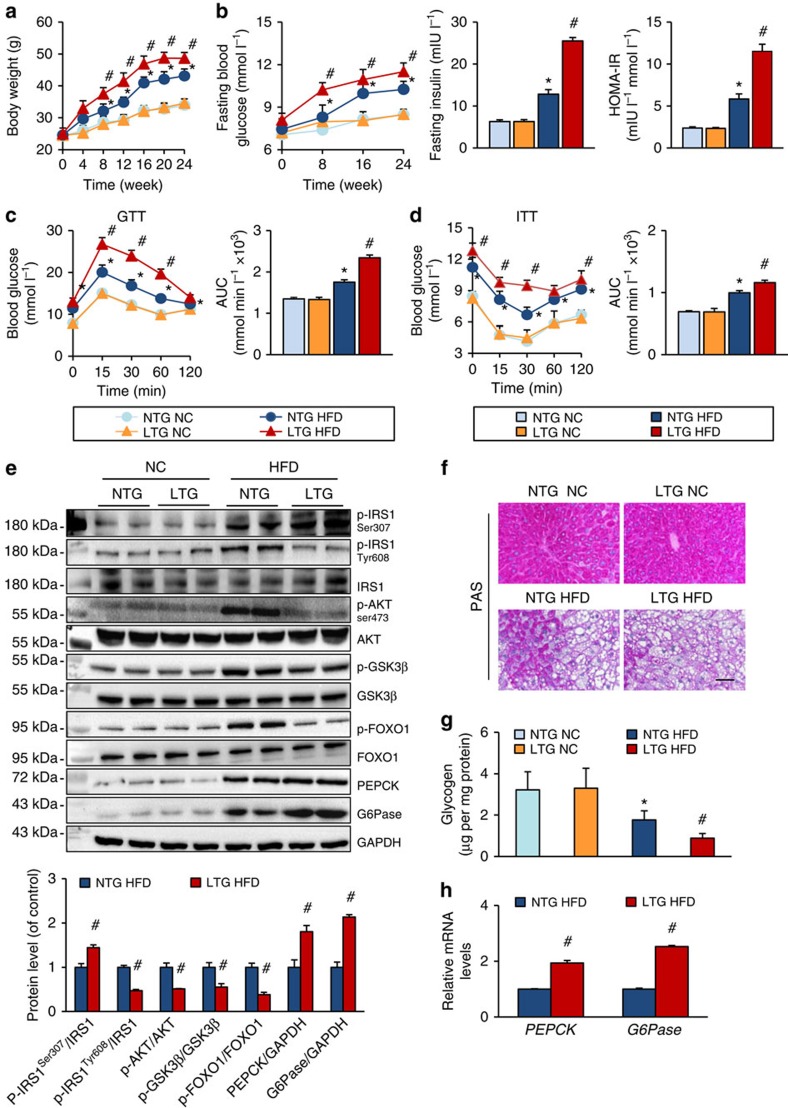
Liver-specific TRAF3 overexpression exacerbates HFD-induced obesity and insulin resistance. (**a**) The body weights of TRAF3-LTG and NTG mice at 0, 4, 8, 12, 16, 20 and 24 weeks after HFD or NC feeding (*n*=17–25 in each group per time point). (**b**) FBG, FINS and HOMA-IR at indicated time points of TRAF3-LTG and NTG mice fed an HFD or NC diet (*n*=10–12 for each group). (**c**,**d**) The intraperitoneal glucose tolerance test (GTT) (**c**) and intraperitoneal insulin tolerance test (ITT) (**d**) assays were performed to evaluate the insulin sensitivity of mice in the indicated groups after HFD or NC treatment for 24 weeks. Area under the curve (AUC) was calculated (*n*=10–12 for each group). (**e**) The expression levels of the key factors in the insulin signalling measured in liver samples of HFD-fed mice that were refed for 4 h after overnight fasting and NC mice under fasting state. (**f**,**g**) The glycogen content levels examined by periodic acid–Schiff (PAS) staining (**f**, *n*=3–4 per group; Scale bar, 50 μm) and Glycogen Assay Kit (**g**, *n*=8–12 per group) on the liver samples of TRAF3-flox and TRAF3-LKO mice after a 24-week HFD or NC treatment. (**h**) *PEPCK* and *G6Pase* expression levels in the liver from mice in the indicated groups measured by RT–PCR analyses. *n*=4 for each group. For **a**–**h**, *P<0.05 versus NTG/NC group; #*P*<0.05 versus NTG/HFD group. All values are means±s.d. Significance determined by two-way analysis of variance with general linear model procedures using a univariate approach (**a**–**d**,**g**) and Student's two-tailed *t*-test (**e**,**h**).

Next, four liver-specific TRAF3-transgenic (TRAF3-LTG) mouse lines were created to further validate the regulatory function of hepatocyte TRAF3 in hepatic steatosis-related obesity and insulin resistance (Supplementary Fig. 3a–c). LTG4 mice were employed for the subsequent investigations. The energy intake was not significantly different in TRAF3-LTG and non-transgenic (NTG) mice after HFD or NC administration (Supplementary Fig. 3d). In contrast to what was observed in TRAF3-LKO mice, the body weight gain and the increase in visceral adipose weight in response to HFD treatment, but not the adipocyte enlargement, were significantly greater in TRAF3-LTG mice compared with NTG controls (Fig. 3a and Supplementary Fig. 4a,b). These effects were accompanied by elevated FBG contents, FINS levels and HOMA-IR (Fig. 3b). The intraperitoneal glucose tolerance test and intraperitoneal insulin tolerance test demonstrated that HFD-induced insulin resistance in NTG mice was further exacerbated by TRAF3 overexpression in the liver (Fig. 3c,d). In addition, the insulin signalling activation was greatly decreased by liver-specific TRAF3 overexpression under HFD refeeding state (Fig. 3e). Consistently, hepatic TRAF3 significantly promoted gluconeogenesis and blocked glycogen synthesis compared with NTG controls (Fig. 3e–h).

In parallel with investigations on the *in vivo* model, the impaired insulin signalling and glycogen synthesis induced by palmitate were ameliorated by TRAF3 deficiency, but aggravated in TRAF3-LTG hepatocytes compared with their corresponding control groups (Supplementary Fig. 5a,b). In addition, gluconeogenesis in primary hepatocytes was increased by TRAF3 after palmitate administration (Supplementary Fig. 5c), whereas hepatocyte TRAF3 showed no observable influence in glucagon-stimulated hepatic glucose production (HGP) and mRNA levels of *PEPCK* and *G6Pase* in primary hepatocytes (Supplementary Fig. 6a,b), although it has been reported that another TRAF member TRAF2 can positively regulate the glucagon-stimulated HGP response^32^. Thus, based on the combined results from the gain- and loss-of-function approaches *in vivo* and *in vitro*, we concluded that the upregulated TRAF3 expression in hepatocytes functions as a positive regulator of HFD-induced obesity and insulin resistance.

### TRAF3 promotes hepatic steatosis and inflammatory response

**Figure 4:**
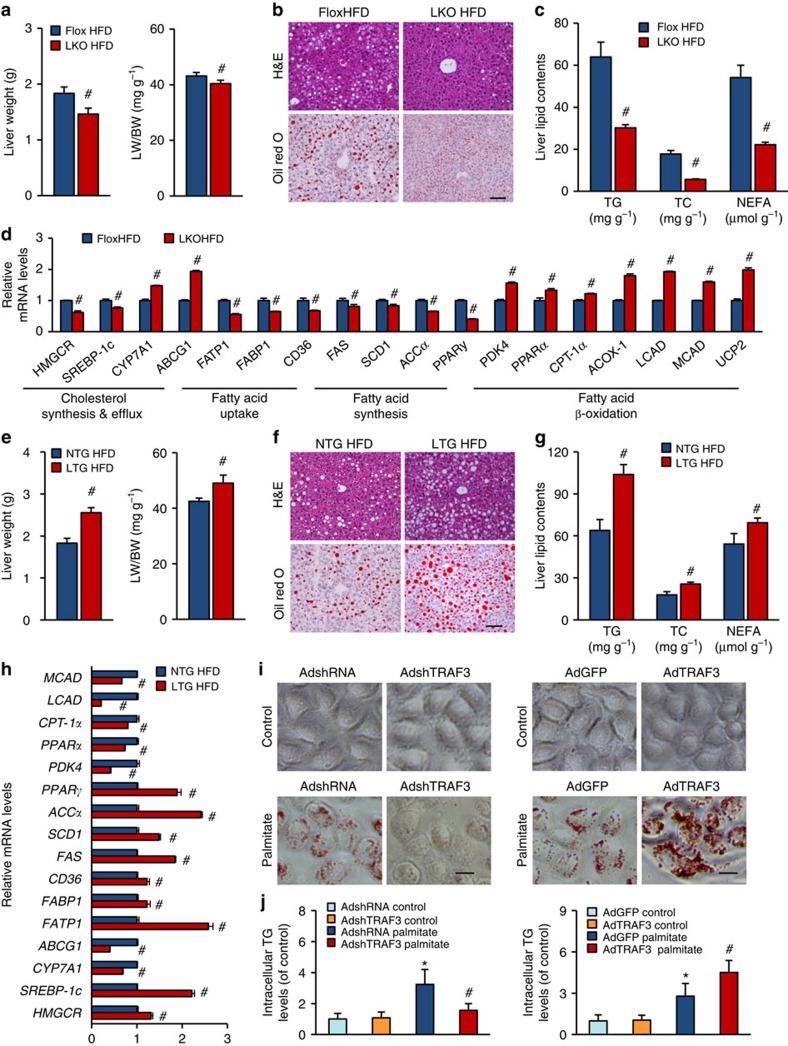
Hepatocyte TRAF3 promotes hepatic steatosis induced by HFD treatment. (**a**,**e**) The liver weights and the ratio of liver weight to body weight (LW/BW) of liver-specific TRAF3-deficient mice and controls (**a**) or of TRAF3-LTG and NTG littermates (**e**) at 24 weeks post-HFD administration (*n*=17–25 samples per experimental group). (**b**,**f**) Haematoxylin and eosin (H&E; upper) and oil red O (bottom) staining of liver sections of TRAF3-LKO and floxed controls (**b**) or TRAF3-LTG and NTG groups (**f**) after HFD treatment (*n*=6–8 samples per experimental group). Scale bar, 100 μm. (**c**,**g**) Hepatic TG, TC and NEFA contents of mice in the indicated groups were measured by enzyme-linked immunosorbent assay (*n*=6–8 samples per experimental group). (**d**,**h**) Quantitative RT–PCR was performed to determine mRNA expressions of genes related to cholesterol synthesis and efflux and fatty acid uptake, synthesis and β-oxidation in the liver samples of TRAF3-flox and TRAF3-LKO mice (**d**) or TRAF3-LTG and NTG mice (**h**) after HFD treatment for 24 weeks (*n*=6–8 samples per experimental group). #*P*<0.05 versus TRAF3-flox/HFD or NTG/HFD group, two-tailed, unpaired Student's *t*-test. (**i**,**j**) Lipid accumulations displayed by oil red O staining (**i**) and intracellular TG assay (**j**) in L-02 cell line infected with AdshTRAF3, AdTRAF3 or their corresponding controls, and subjected to palmitate or vehicle control administration for 24 h (*n*=10 samples per experimental group). Scale bar, 10 μm. *P<0.05 versus AdGFP/AdshRNA control group; #*P*<0.05 versus AdGFP/AdshRNA palmitate group; significance determined by two-way analysis of variance with general linear model procedures using a univariate approach.

A continuous excess energy intake can lead to the over-accumulation of lipid in the livers that is often observed in obese individuals and can be exacerbated by insulin resistance^24^. After a 24-week HFD treatment, the liver size, liver weight and ratio of liver weight to body weight were considerably reduced in TRAF3-LKO mice compared with the floxed control group (Supplementary Fig. 7 and Fig. 4a). Consistent with these findings, haematoxylin and eosin and oil red O staining showed markedly fewer lipid droplets in the liver of TRAF3-deficient mice than in TRAF3-flox controls after 24 weeks of HFD administration (Fig. 4b). As shown in Fig. 4c, the contents of triglycerides (TGs), total cholesterol (TC) and non-esterified fatty acids (NEFAs) in the livers of TRAF3-LKO mice were obviously reduced compared with TRAF3-flox controls after HFD treatment for 24 weeks. Meanwhile, the liver dysfunction induced by HFD was suppressed by TRAF3 ablation, as shown by the markedly decreased serum alanine amino transferase (ALT), aspartate amino transferase (AST) and alkaline phosphatase (ALP) levels in liver-specific TRAF3-LKO mice (Supplementary Table 1). Quantitative real-time–PCR (RT–PCR) was performed to examine the expression levels of genes related to fatty acid synthesis and elimination. The expression of genes related to cholesterol synthesis (3-hydroxy-3-methylglutaryl CoA reductase (*HMGCR*) and sterol regulatory element-binding protein-1c (*SREBP-1c*)), fatty acid uptake (*CD36*, fatty acid transport protein 1 (*FATP1*) and fatty acid-binding protein 1 (*FABP1*)) and fatty acid synthesis (fatty acid synthase (*FAS*), stearoyl-CoA desaturase-1 (*SCD1*), acetyl CoA carboxylase α (*ACCα*) and peroxisome proliferator-activated receptor gamma (*PPARγ*)) was significantly inhibited by TRAF3 deficiency, whereas the expression of genes responsible for cholesterol efflux (cholesterol 7α-hydroxylase (*CYP7A*) and ATP-binding cassette transporter G1 (*ABCG1*)) and fatty acid β-oxidation (pyruvate dehydrogenase kinase 4 (*PDK4*), *PPARα*, and carnitine palmitoyltransferase 1 (*CPT-1α*)) was much higher in TRAF3-LKO mice than in TRAF3-flox mice after HFD treatment for 24 weeks (Fig. 4d).

In contrast to amelioration of hepatic steatosis in TRAF3-LKO mice, the liver-specific overexpression of TRAF3 caused an opposite effect on hepatic lipid accumulation and liver function. As shown in Fig. 4e, the liver was heavier in the TRAF3-LTG group than in NTG controls after a 24-week HFD administration, which might be attributed to increased lipid deposition in the liver (Fig. 4f,g). The examination of ALT, AST and ALP serum levels further confirmed the detrimental effect of hepatocyte TRAF3 upregulation on liver function (Supplementary Table 1). Moreover, PCR analysis results demonstrated that liver-specific TRAF3 overexpression markedly enhanced cholesterol synthesis, fatty acid uptake and fatty acid synthesis, but inhibited cholesterol export and fatty acid decomposition (Fig. 4h). Consistent with the *in vivo* investigations into HFD-induced hepatic steatosis, TRAF3's function in exacerbating lipid accumulation was found in L-02 hepatocyte cell line treated with palmitate as shown by oil red O staining (Fig. 4i). Measurements of the intracellular TG levels in L-02 hepatocytes also showed that after palmitate administration, the lipid content was significantly reduced by TRAF3 knockdown (AdshTRAF3) but enhanced by TRAF3 overexpression (AdTRAF3) compared with AdshRNA or AdGFP groups (Fig. 4j).

**Figure 5:**
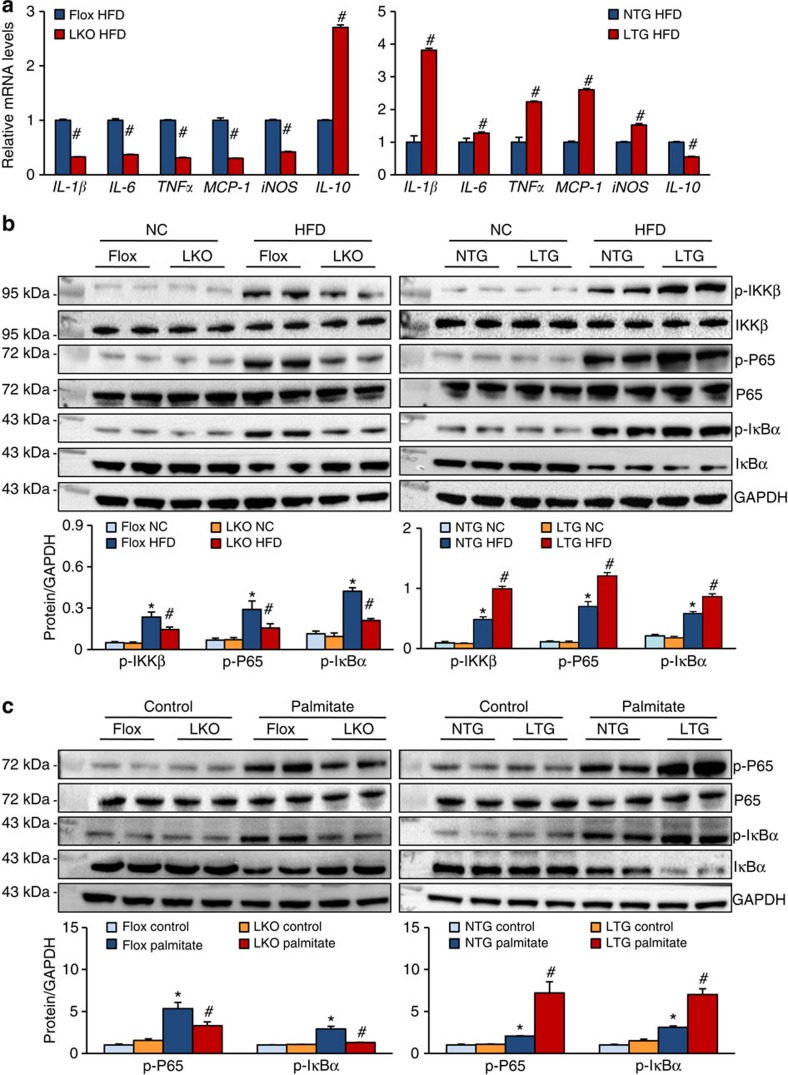
HFD-induced hepatic inflammatory response can be significantly aggravated by TRAF3 in hepatocytes. (**a**) mRNA expression levels of inflammatory cytokines and chemokines in the liver of indicated groups (*n*=6–8 samples per experimental group). (**b**) The expression levels of key proteins in NF-κB signalling in the liver of TRAF3-LKO, TRAF3-LTG and their corresponding littermate controls after HFD or NC treatment for 24 weeks. Protein expression was normalized to that of GAPDH (*n*=4 samples per experimental group). For **a** and **b**, *P<0.05 versus TRAF3-flox/NC or NTG/NC group; #*P*<0.05 versus TRAF3-flox/HFD or NTG/HFD group. (**c**) Primary hepatocytes were isolated from TRAF3-LKO, TRAF3-LTG, TRAF3-flox and NTG groups followed by stimulation with palmitate (0.25 mM) or vehicle for 24 h. Total and phosphorylated p65 and IκBα were detected by western blot analysis. GAPDH served as a loading control (*n*=6 samples per experimental group). *P<0.05 versus TRAF3-flox/control or NTG/control group; #*P*<0.05 versus TRAF3-flox/palmitate or NTG/palmitate group. All values are means±s.d. Significance determined by Student's two-tailed *t*-test (**a**) and two-way analysis of variance with general linear model procedures using a univariate approach (**b**,**c**).

In addition to its enhancement of hepatic lipid accumulation, TRAF3 also increased the amount of HFD-induced abnormal lipid in the serum. As shown in Supplementary Fig. 8a, serum levels of TG, TC, low-density lipoprotein and free fatty acid were lower, but the HDL level was higher, in TRAF3-LKO mice than those in TRAF3-flox controls after chronic HFD feeding. In contrast, TRAF3-LTG had the opposite effect on serum lipid levels. The expression levels of inflammatory mediators in the serum and liver samples were measured, for that NAFLD is closely associated with a chronic inflammatory response^5^,^24^. The serum levels of pro-inflammatory cytokines (for example, interleukin (IL)-1β, IL-4, IL-6, TNF-α and IL-2) and chemokines monocyte chemotactic protein-1 (MCP-1) were significantly lower in TRAF3-LKO mice, but higher in TRAF3-LTG mice than in the corresponding controls after HFD treatment (Supplementary Fig. 8b). Conversely, the production of anti-inflammatory mediator IL-10 in the serum was inhibited by TRAF3 (Supplementary Fig. 8b). TRAF3 consistently influenced the expression of genes encoding inflammatory factors in the liver samples from the indicated groups (Fig. 5a). In addition, canonical NF-κB signalling was activated by HFD treatment *in vivo* and by palmitate challenge *in vitro*. This signalling was further enhanced by TRAF3 overexpression (Fig. 5b,c). However, the level of p52/RelB protein in the nucleus was not significantly affected by TRAF3, although numerous studies indicated that TRAF3 potently regulates the alternative NF-κB pathway cascade^33^ (Supplementary Fig. 9). Collectively, these findings show that hepatocyte TRAF3 exacerbates HFD-induced hepatic steatosis and the related inflammatory responses.

In addition to the excess energy intake model, an *ob/ob* (leptin-deficient) mouse model was employed in this study to evaluate the role of hepatic TRAF3 in genetically induced hepatic steatosis. Supplementary Fig. 10a showed that TRAF3 level was higher in the livers of *ob/ob* mice than in those of lean controls, and the knockdown of TRAF3 by AdshTRAF3 injection significantly inhibited the spontaneous insulin resistance, glucose intolerance, hepatic steatosis and inflammatory response in these mice (Supplementary Fig. 10b–k). Thus, it can be concluded that HFD-induced and genetically induced insulin resistance, inflammatory response and hepatic steatosis are negatively regulated by TRAF3 deficiency.

### TRAF3 regulates hepatic steatosis dependent on TAK1–JNK axis

**Figure 6:**
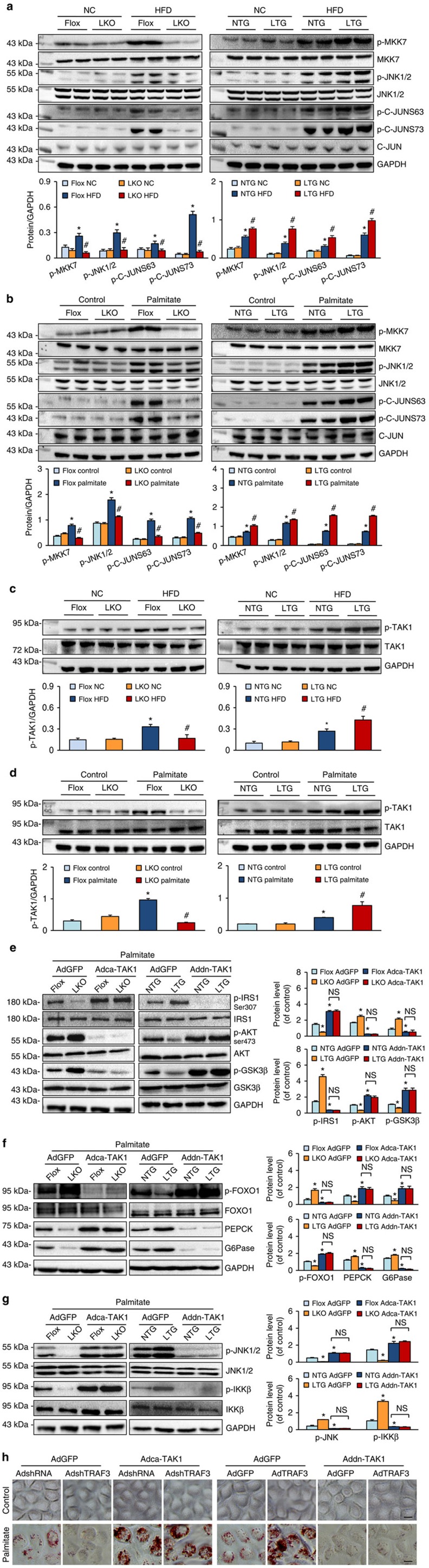
TAK1–JNK signalling is involved in TRAF3-regulated hepatic steatosis. (**a**) MKK–JNK signalling activation was measured from the total and phosphorylated MKK7, JNK and C-JUN levels in the livers of TRAF3-LKO mice, TRAF3-LTG mice and their control littermates after HFD treatment for 24 weeks (*n*=6 samples per experimental group). (**b**) The expression of proteins in the MKK–JNK–C-JUN cascades in primary hepatocytes isolated from mice in the indicated groups and subjected to palmitate stimulation (0.25 mM) for 24 h (*n*=6 samples per experimental group). (**c**) TAK1 and p-TAK1 expression in the liver of TRAF3-flox, TRAF3-LKO, NTG and TRAF3-LTG mice at 24 weeks after HFD or NC administration (*n*=6 samples per experimental group). (**d**) TAK1 and p-TAK1 expression in primary hepatocytes in the indicated groups after palmitate (0.25 mM) or vehicle treatment for 24 h (*n*=6 samples per experimental group). Protein expressions were normalized to that of GAPDH. For **a** and **c**, *P<0.05 compared with TRAF3-flox/NC or NTG/NC group; #*P*<0.05 compared with TRAF3-flox/HFD or NTG/HFD group. For **b** and **d**, *P<0.05 versus TRAF3-flox/control or NTG/control group; #*P*<0.05 compared with TRAF3-flox/palmitate or NTG/palmitate group. (**e**–**g**) Total and/or phosphorylated protein levels of key factors in the insulin signalling (**e**), gluconeogenesis-related axis (**f**) and JNK/IKKβ expression (**g**) in Adca-TAK1-infected primary TRAF3-flox or TRAF3-LKO hepatocytes challenged by palmitate, or in Addn-TAK1-treated hepatocytes isolated from NTG or TRAF3-LTG mice. Primary hepatocytes infected with AdGFP were served as controls. *n*=4 samples per experimental group. NS, no significant difference; *P<0.05 versus TRAF3-flox/AdGFP or NTG/AdGPF group. (**h**) Representative images of oil red O-stained L-02 cells co-infected with AdshTRAF3 and Adca-TAK1, or with AdTRAF3 and Addn-TAK1, and stimulated with palmitate for 24 h. AdGFP- or AdshRNA-infected cells were served as controls. Scale bar, 10 μm. All values are means±s.d. Significance determined by two-way analysis of variance with general linear model procedures using a univariate approach.

The potent regulatory activity of TRAF3 in hepatic steatosis and associated pathologies prompted us to explore the underlying mechanisms. Considering the involvement of MAPKs, particularly JNK and p38 MAPK cascades, in the progression of this pathological condition and the capacity of TRAF3 to regulate MAPK activation^16^,^24^,^34^, the levels of total and phosphorylated protein in this signalling were measured. Western blotting results revealed that ERK, p38 and JNK signalling were simultaneously activated in the liver in response to HFD treatment (Supplementary Fig. 11 and Fig. 6a), whereas TRAF3 only markedly enhanced the activation of the MKK7–JNK–C-JUN pathway induced by HFD *in vivo* or palmitate *in vitro* (Fig. 6a,b); no significant influence in the level of phosphorylated MEK, ERK or p38 by TRAF3 was observed (Supplementary Fig. 11). Moreover, the activation of transforming growth factor-β-activated kinase 1 (TAK1), a crucial upstream factor of JNK signalling, was significantly increased by TRAF3 overexpression but inhibited by the ablation of TRAF3 in the liver after HFD or palmitate challenge (Fig. 6c,d). These data collectively demonstrated that the TAK1–JNK axis pathway is closely regulated by TRAF3 during the progression of hepatic steatosis.

Next, we explored whether the presence of TAK1 is required for the functional regulation of hepatocyte TRAF3 through infecting primary hepatocytes isolated from TRAF3-LKO, TRAF3-LTG and their corresponding control mice with Adca-TAK1, Addn-TAK1 or AdGFP in parallel. After palmitate administration, Adca-TAK1 markedly blunted insulin signalling in both TRAF3-flox and TRAF3-LKO cells, whereas the TRAF3 overexpression-induced impairment of insulin signalling was largely reversed by Addn-TAK1 infection in primary hepatocytes (Fig. 6e). Figure 6f displayed that the gluconeogenesis-related protein levels were consistently increased in TRAF3-flox and TRAF3-LKO hepatocytes by Adca-TAK1 administration, and that PEPCK and G6Pase expression levels were decreased by Addn-TAK1 after palmitate challenge. In addition, TAK1 significantly enhanced the JNK1/2 and IKKβ activations (Fig. 6g). Oil red O staining showed that artificial TAK1 upregulation abolished the regulatory effects of TRAF3 deficiency, and TAK1 downregulation reversed the role of TRAF3 overexpression, in palmitate-induced fatty acid accumulation (Fig. 6h). The respective influences of Adca-TAK1 and Addn-TAK1 on TRAF3 deficiency- and overexpression-regulated lipid metabolism were also confirmed by the RT–PCR analyses (Supplementary Fig. 12). Thus, TAK1 is required for TRAF3-regulated aggravation of lipid metabolism in primary hepatocytes on palmitate challenge.

**Figure 7:**
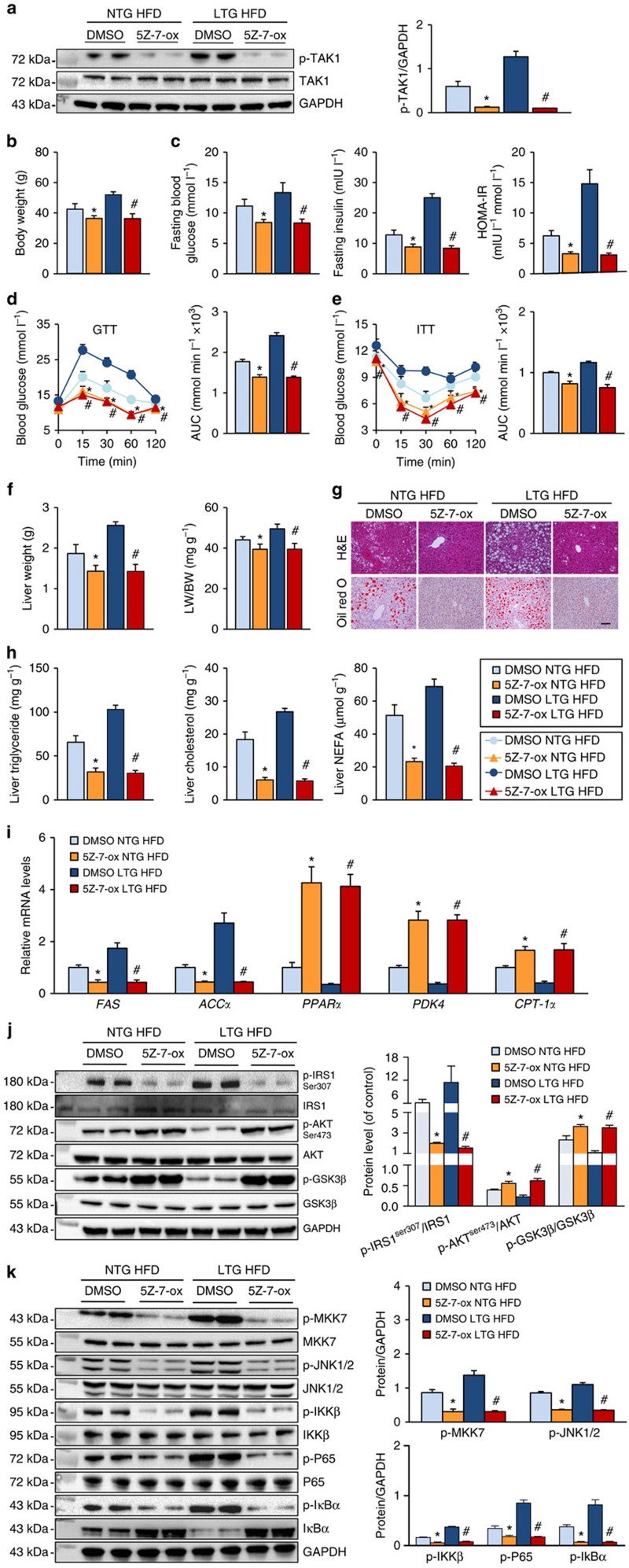
Hepatocyte TRAF3 regulates HFD-induced obesity, insulin resistance and hepatic steatosis in a TAK1-dependent manner. (**a**) Identification of the inhibitory effect of 5Z-7-ox on TAK1 activation by western blotting (*n*=6 samples per experimental group). 5Z-7-ox was administered intraperitoneally to NTG and TRAF3-LTG mice weekly at 5 mg kg^−1^ and continued for 24 weeks. (**b**) The body weights of NTG and TRAF3-LTG mice treated with or without 5Z-7-ox at 24 weeks after HFD administration (*n*=16–22). (**c**) FBG, FINS and HOMA-IR levels in mice with liver-specific TRAF3 overexpression and NTG littermates after 5Z-7-ox injection at 24 weeks post-HFD treatment (*n*=8–10). (**d**,**e**) Intraperitoneal glucose tolerance test (GTT) (**d**) and intraperitoneal insulin tolerance test (ITT) (**e**) were performed on the mice treated with a TAK1 inhibitor after continuous HFD feeding for 24 weeks, and the area under the curve (AUC) of glucose levels was measured (*n*=8–10). (**f**) The liver weight and the ratio of liver weight to body weight (LW/BW) of mice in the indicated groups after HFD administration for 24 weeks (*n*=16–22). (**g**) Representative haematoxylin and eosin (H&E; upper)- and oil red O (bottom)-staining images of liver sections from mice in the indicated group treated with HFD for 24 weeks (*n*=6–8). Scale bar, 100 μm. (**h**) TG, TC and NEFA levels in the liver samples of NTG and TRAF3-LTG mice injected with 5Z-7-ox at 24 week after HFD administration (*n*=6–8). (**i**) Relative mRNA expression of genes closely associated with fatty acid metabolism in the liver samples from mice in indicated groups after a 24-week HFD administration. (**j**) Total and phosphorylated IRS1, AKT and GSK3β levels in the liver samples from indicated mice with or without 5Z-7-ox treatment 24 weeks after HFD feeding (*n*=6 samples per experimental group). (**k**) The expression of proteins in the NF-κB and JNK signalling pathways in the liver samples of mice in the indicated groups after HFD administration for 24 weeks (*n*=6 samples per experimental group). GAPDH served as a loading control. *P<0.05 versus NTG/DMSO group; #*P*<0.05 versus TRAF3-LTG/DMSO group. All values are means±s.d. Significance determined by two-way analysis of variance with general linear model procedures using a univariate approach.

To further evaluate the requirement for TAK1 activation during TRAF3-regulated hepatic steatosis *in vivo*, the TAK1 inhibitor 5Z-7-ox was intraperitoneally (i.p.) administered to NTG and TRAF3-LTG mice, and the inhibitory efficacy of this compound was confirmed by the reduced phosphorylation of TAK1 (Fig. 7a). The increased body weight gain and impaired insulin sensitivity induced by HFD were markedly reduced by 5Z-7-ox treatment in NTG mice. More importantly, this TAK1 inhibitor largely abolished the exacerbation of obesity and insulin resistance that was observed in mice with liver-specific TRAF3 overexpression (Fig. 7b–e). Moreover, the increased liver weight, enhanced lipid accumulation and exacerbated disturbance of fatty acid metabolism-related gene expression profiles in TRAF3-LTG mice were almost completely reversed by 5Z-7-ox administration (Fig. 7f–i). As expected, the inhibition of TAK1 significantly blocked the phosphorylation of IRS at Ser307, but increased the phosphorylated expression of AKT and GSK3β, in both NTG and TRAF3-LTG mice (Fig. 7j), and the HFD-triggered activation of MKK–JNK and NF-κB signalling was markedly inhibited by 5Z-7-ox treatment *in vivo* (Fig. 7k). Therefore, the deleterious effects of TRAF3 on hepatic steatosis are largely dependent on the activation of TAK1.

### The interaction between TRAF3 and TAK1

**Figure 8:**
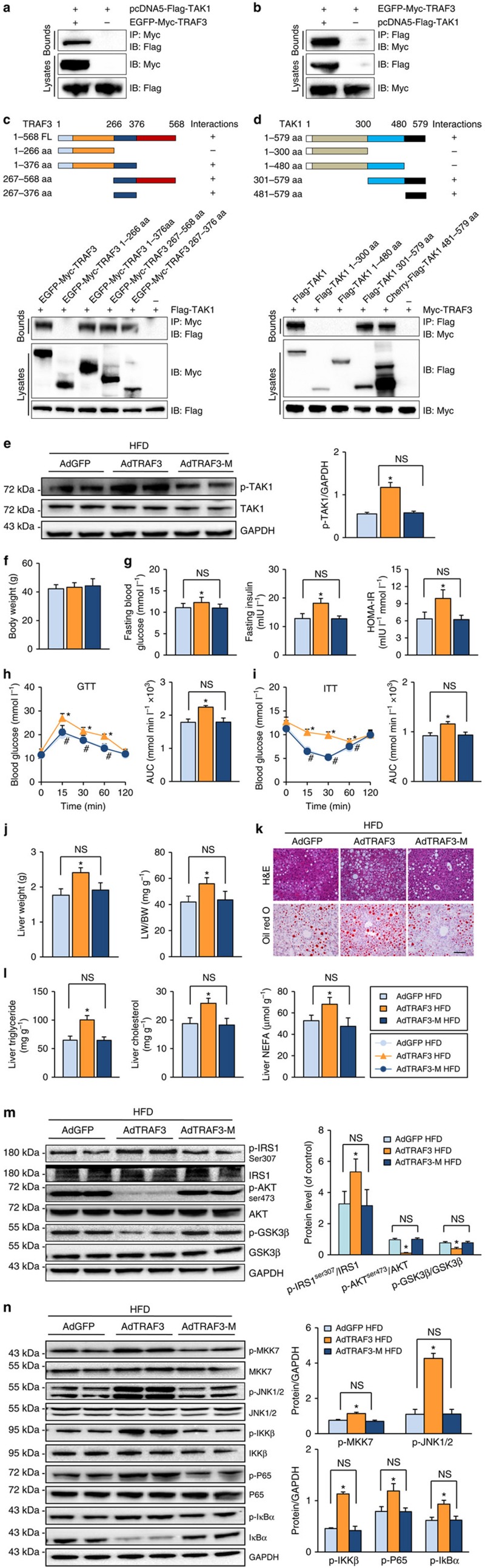
The TRAF3–TAK1 interaction is required for TRAF3-regulated hepatic steatosis and insulin resistance. (**a**,**b**) A co-immunoprecipitation assay was performed to examine the interaction of TRAF3 with TAK1. (**c**,**d**) The interaction domains of TRAF3 and TAK1 were explored using full-length and truncated TRAF3 (**c**) and TAK1 (**d**) expression constructs based on co-immunoprecipitation assays. Anti-Myc or anti-Flag antibodies were used to identify the binding sites of TRAF3 and TAK1, respectively. (**e**) WT mice were infected with an adenoviral vector carrying TRAF3 (AdTRAF3) or a TRAF3 mutant with 267–376 aa domain mutation (AdTRAF3-M) at 20 weeks after HFD treatment, and TAK1 and p-TAK1 expression was measured by western blot after an additional 4 weeks. Mice injected with AdGFP served as controls. Protein expression levels were normalized to that of GAPDH (*n*=6 samples per experimental group). (**f**) The body weights of mice injected with AdGFP, AdTRAF3 or AdTRAF3-M after an additional 4 weeks' HFD treatment (*n*=17–25). (**g**) FBG and FINS levels were examined in indicated mice post-HFD administration for an additional 4 weeks, and HOMA-IR was calculated (*n*=6–8). (**h**,**i**) Intraperitoneal glucose tolerance test (GTT) (**h**) and intraperitoneal insulin tolerance test (ITT) (**i**) were performed on the mice in the indicated groups after 4 weeks' HFD treatment. The area under the curve (AUC) of glucose levels was measured (*n*=6–8). (**j**) The liver weight and ratio of liver weight to body weight (LW/BW) value of indicated mice after 4 weeks' HFD treatment (*n*=17–25). (**k**) Images of haematoxylin and eosin (H&E; upper) or oil red O (bottom) staining of liver sections of mice injected with indicated adenovirus (*n*=6–8). Scale bar, 100 μm. (**l**) Liver TG, TC and NEFA levels in indicated mice fed an HFD for an additional 4 weeks (*n*=6–8). (**m**,**n**) The expression levels of proteins in the IRS–AKT–GSK3β insulin signalling (**m**) as well as NF-κB and JNK signalling pathways (**n**) in the liver samples from indicated mice. Protein expression was examined by western blotting and normalized to the indicated total protein or GAPDH expression after 4 weeks' HFD treatment (*n*=6 samples per experimental group). For **g**–**n**, *P<0.05 versus AdGFP group; NS, no significant difference. All values are means±s.d. Significance determined by two-way analysis of variance with general linear model procedures using a univariate approach.

The essential role of TAK1 in TRAF3-regulated hepatic steatosis and insulin resistance raised another critical question: do TRAF3 and TAK1 directly interact during the progression of this pathological condition? To answer this question, immunoprecipitation assays were performed. The results suggested that exogenetic expression of TRAF3 could interact with TAK1 and *vice versa* (Fig. 8a,b). Further co-immunoprecipitation experiments using a series of truncated Myc-TRAF3 and TAK1 mutants indicated that the 267–376 amino acid (aa) domain (TRAF-N) of TRAF3 and the 481–579 aa domain (TAB2/3 BD) of TAK1, respectively, contributes to their capacity to bind to each other (Fig. 8c,d). Next, to evaluate whether TRAF3–TAK1 interaction is required for the regulatory function of TRAF3 on hepatic steatosis, an adenovirus harbouring TRAF3 with a mutant 267–376 aa domain (AdTRAF3-M) was constructed and injected into WT mice in parallel with AdTRAF3 at 20 weeks of HFD administration. Mice injected with AdGFP served as controls. As shown in Fig. 8e, AdTRAF3-M failed to significantly increase the expression of p-TAK1 in the liver. After further HFD treatment for 4 weeks, although HFD-induced obesity was not significantly influenced by TRAF3 upregulation (Fig. 8f), mice with AdTRAF3 injection clearly exhibited increased fasting glucose and insulin levels but decreased insulin sensitivity and glucose tolerance (Fig. 8g–i). However, the AdTRAF3-M treatment rendered comparable influence in obesity and insulin resistance with that of AdGFP administration (Fig. 8g–i). In addition, the injection of AdTRAF3, but not the injection of AdTRAF3-M, increased lipid accumulation in the liver and the resulting increase in liver weight (Fig. 8j–l). Western blot analyses demonstrated that the capacity of TRAF3 to inhibit insulin signalling and to promote the MKK–JNK and NF-κB cascades was nullified by the impairment of TRAF3–TAK1 interaction (Fig. 8m,n). On the basis of these experiments, we deduced that the proposed interaction between TRAF3 and TAK1 at least partially explain the regulatory function of TRAF3 on hepatic steatosis and insulin resistance.

### TRAF3 activates TAK1 through ubiquitination

**Figure 9:**
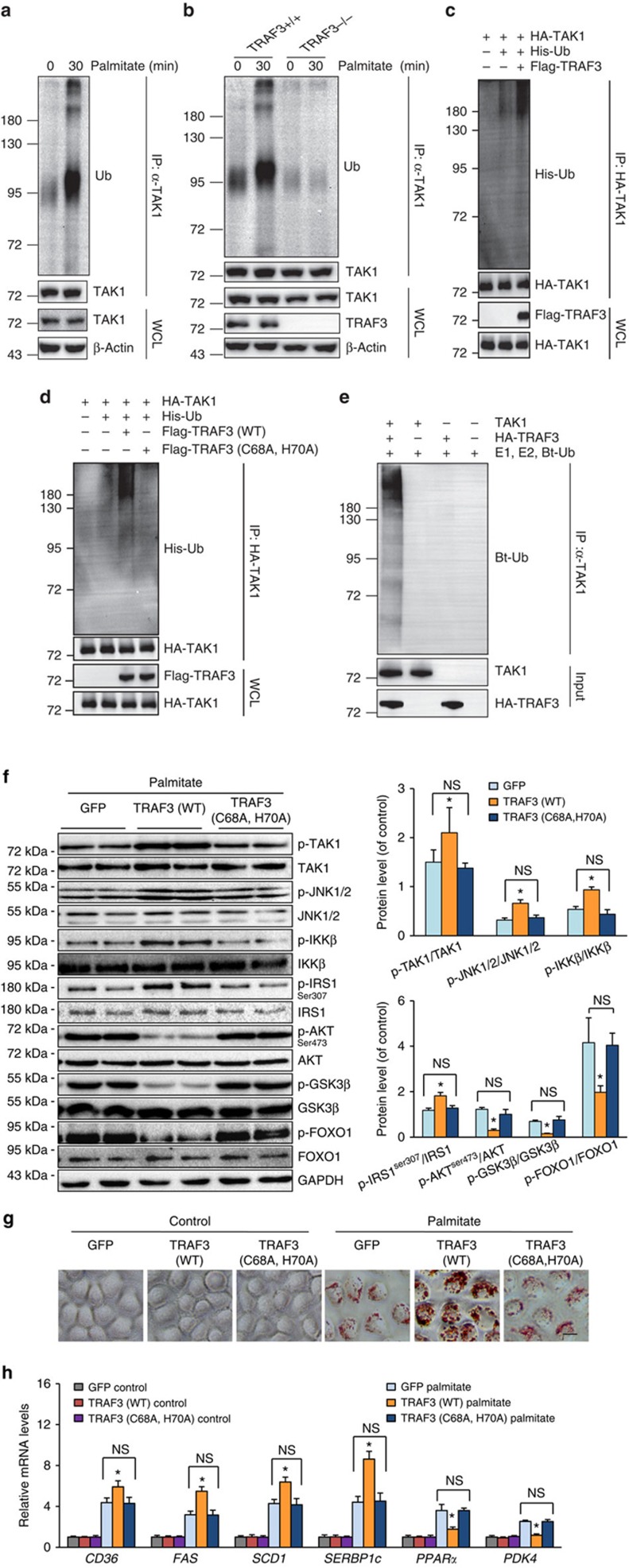
TRAF3 regulates lipid accumulation in hepatocytes dependent on TAK1 ubiquitination. (**a**) The ubiquitination of TAK1 in palmitate-stimulated primary WT hepatocytes was examined using immunoprecipitation with an anti-TAK1 antibody followed by western blotting with an anti-Ub antibody. (**b**) Immunoprecipitation assay measured the TAK1 ubiquitination in primary hepatocytes isolated from TRAF3^+/+^ and TRAF3^−/−^ mice, and stimulated with palmitate for 30 min. 293T cells were transfected with HA-TAK1 and His-Ub along with/without the infection of Flag-TRAF3 (**c**) or Flag-TRAF3 (C68A, H70A) (**d**), and subjected to immunoprecipitation and western blotting assays. (**e**) *In vitro* TAK1 ubiquitination was measured through incubating TAK1 protein with E1, E2 and biotinylated-Ub (Bt-Ub) combined with or without HA-TRAF3 protein and the subsequent immunoprecipitation and western blot analyses. (**f**) Expression levels of key proteins in the TAK1–JNK/IKKβ cascades and insulin signalling were examined by western blotting in primary hepatocytes overexpressed TRAF3 (WT) or TRAF3 (C68A, H70A) and treated with palmitate for 24 h. Primary hepatocytes infected with GFP were as controls. GAPDH serves as loading controls (*n*=6 samples per experimental group). (**g**) Representative oil red O staining on the L-02 cells infected with AdGFP, AdTRAF3 (WT) or AdTRAF3 (C68A, H70A) after palmitate stimulation for 24 h. Scale bar, 10 μm. (**h**) The mRNA expression of genes related with lipid metabolisms in palmitate- or vehicle solution-treated L-02 cells overexpressed TRAF3 (WT), TRAF3 (C68A, H70A) or GFP. For **f**,**h**, NS, no significant difference; *P<0.05 versus GFP/palmitate group. All values are means±s.d. Significance determined by two-way analysis of variance with general linear model procedures using a univariate approach.

Observations of the direct binding of TRAF3 to TAK1 and the increased TAK1 phosphorylation during TRAF3-regulated hepatic steatosis promoted us to investigate the process occurring between TRAF3–TAK1 interaction and TAK1 activation. Considering the necessity of ubiquitination for the TAK1 autophosphorylation/activation and the K63-polyubiquitination catalysis ability of TRAF3 (refs 35, 36, 37), we examined whether an involvement of TAK1 ubiquitination exists in the TRAF3-regulated development of fatty acid metabolic disorder. Figure 9a showed that ubiquitination of TAK1 was markedly induced by palmitate stimulation (30 min) in primary hepatocytes isolated from WT mice. The ubiquitination of TAK1 was abolished when TRAF3 is absent (Fig. 9b). Studies on the 293T cell line transfected with HA-TAK1 with or without Flag-TRAF3 plasmids further confirmed the potentiated effect of TRAF3 on TAK1 ubiquitination (Fig. 9c); however, after blocking the E3 ubiquitin ligase activity (C68A, H70A), TRAF3 lost its capacity to enhance TAK1 ubiquitination (Fig. 9d). To provide direct evidence for the ubiquitin-mediated activation of TAK1 by TRAF3, TAK1 protein was co-incubated with E1, E2 and biotinylated-Ub in the presence of or absence of HA-TRAF3, with the result that TRAF3 can directly induce TAK1 ubiquitination *in vitro* (Fig. 9e).

To examine whether TRAF3-induced TAK1 ubiquitination is essential for TRAF3-regulated hepatic steatosis, adenovirus carrying a catalytically inactive TRAF3 (C68A, H70A) mutant was employed to infect primary hepatocytes that were subjected to palmitate challenge. Western blotting indicated that the TRAF3 mutation did not influence insulin signalling and TAK1–JNK/NF-κB pathways, unlike in TRAF3 (WT)-treated cells, and had a comparable effect on these molecular events to AdGFP infection (Fig. 9f). Consistent with this finding, oil red O staining and RT–PCR analyses showed that the TRAF3 (C68A, H70A) mutant cannot significantly regulate lipid accumulation or the related fatty acid metabolic gene expression levels in hepatocytes on palmitate stimulation (Fig. 9g,h). Collectively, these observations clearly demonstrated that TRAF3 directly promotes TAK1 ubiquitination, which is required for the regulatory function of TRAF3 in lipid metabolism in hepatocytes.

## Discussion

**Figure 10:**
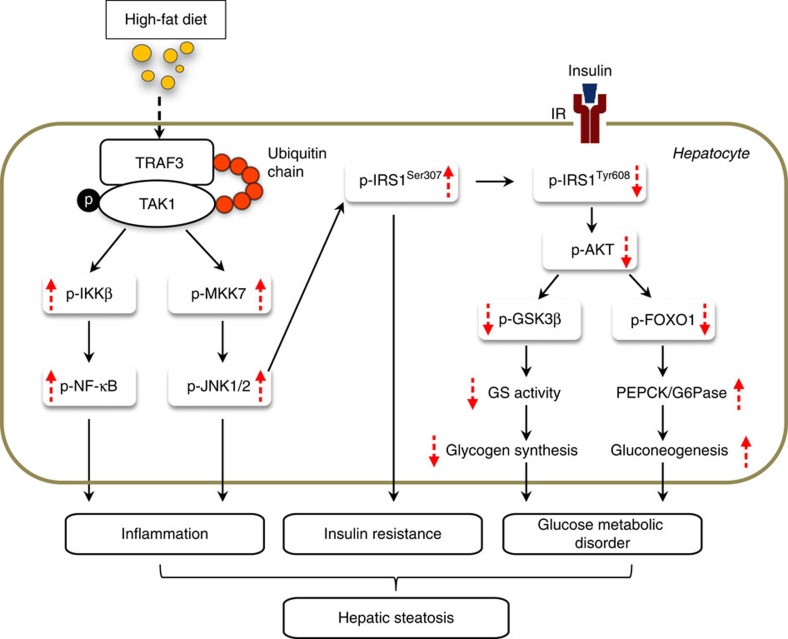
Schematic illustration of cellular and molecular events underlying hepatocyte TRAF3-regulated hepatic steatosis. In response to a continuous challenge with HFD, TRAF3 expression is increased in hepatocytes and directly interact with TAK1 to ubiquinate and phosphorylate TAK1, leading to the activation of downstream IKKβ–NF-κB and MKK–JNK signalling, thereby promoting inflammatory response. The activated JNK further aggravates insulin resistance through enhancing IRS phosphorylation at Ser307 and disrupts its downstream AKT–GSK3β/FOXO1 phosphorylation, which contribute to glucose metabolic disorder. Inflammation and insulin resistance, together with glucose metabolic disorder, synergistically exacerbate fatty acid accumulation and the resultant hepatic steatosis.

In the present study, using liver-specific TRAF3-KO/-transgenic mice, we identified hepatocyte TRAF3 as a positive regulator of HFD-induced or genetically induced insulin resistance, inflammatory responses and hepatic steatosis. Investigations of the underlying mechanisms demonstrated that after a continuous challenge with an HFD, the expression of TRAF3 is upregulated in hepatocytes and binds to and ubiquitinates TAK1, which leads to the autophosphorylation and activation of TAK1. The activated TAK1 further lead to an activation of downstream MKK–JNK–IRS1^307^ and IKKβ–NF-κB signalling, and an inhibition of AKT–GSK3β/FOXO1 phosphorylation cascades, facilitating the insulin resistance, inflammatory response and glucose metabolic disorder in the liver, thereby exacerbating fatty acid accumulation and the resultant hepatic steatosis (Fig. 10).

Hepatic steatosis, insulin resistance and inflammatory response are mutually promoted pathological events under the condition of metabolic disorder^5^. Chronic over-nutrition administration can elicit insulin resistance in the liver, where the normal function of insulin to inhibit glucose production is diminished, but the stimulatory effect of insulin on lipogenesis is retained^38^. Consequently, the development of escalating insulin resistance leads to aberrant lipid accumulation in the liver^39^,^40^. Meanwhile, inflammatory response can be also induced by the excess nutrition, such as HFD, and participates to the onset of insulin resistance^41^. In this study, hepatic TRAF3 expression can be elevated by HFD and in turn promote all of the three pathologies, suggesting a therapeutic promising of TRAF3 for hepatic steatosis-related metabolic diseases. Interestingly, the function of hepatocyte TRAF3 is not only limited in the hepatic events but also expanded to the whole system, including obesity, visceral fat weight gain, circulating lipid disorder and serum inflammatory factor imbalance, which might be the secondary effects of hepatic modulation by TRAF3. However, the molecular events leading to the upregulation of TRAF3 in response to a HFD remain to be determined.

Among the complex network coordinating hepatic lipid metabolism, TAK1, a MAPK kinase kinase (MAP3K) family member, is a prominent factor based on its common regulatory ability on IKKβ–NF-κB and MKK–JNK pathways, the dominant molecular signalling orchestrating inflammation and insulin resistance^42^. It has been well documented that insulin function is synergistically coordinated by IRS-related molecular events^1^,^43^,^44^. After activated by TAK1, JNK1 potently promotes IRS1 phosphorylation at serine307 and subsequently induces the inhibition of IRS1 tyrosine phosphorylation, which leads to compromised capacity of IRS1 to bind to insulin receptors and reduced AKT phosphorylation^45^,^46^. This in turn inhibits glycogen synthesis by reducing the phosphorylation of GSK3β and stimulates gluconeogenesis through enhancing the activation of FOXO1–PEPCK/G6Pase axis^27^,^47^. In contrast to JNK1, the JNK2 isoform shows no direct function in insulin resistance or obesity induced by diet, but JNK2 has a prominent influence in JNK1's activation^48^. Thus, the balance between JNK1 and JNK2 forms a determinant of the pathogenesis of insulin resistance and hepatic steatosis. In this study, the activation of TAK1–MKK7–JNK1/2 and FOXO1–PEPCK/G6Pase cascades was markedly enhanced, but IRS–AKT insulin signalling was inhibited, by TRAF3 overexpression in hepatocytes. On the basis of previous studies and our present findings, it can be deduced that the enhanced TAK1–MKK–JNK activation by hepatic TRAF3 might be, at least partly, responsible for the TRAF3-regulated exacerbation of insulin dysfunction, glucose metabolic disorder and fatty acid accumulation in the liver. However, it has also been reported that TRAF3 negatively regulates phosphorylation of JNK in the downstream of CD40 and TLR signalling through repressing TAK1 phosphorylation^49^,^50^. The paradox of TRAF3-regulated TAK1–JNK activation remains to be further elucidated, but the distribution of TAK1–JNK cascade in diverse cell types and the difference in extracellular stresses might provide a potential explanation.

In addition to its tight association with insulin function, hepatic steatosis is closely related to inflammation and is even defined as a chronic low-grade inflammatory response. The TAK1 downstream IKKβ–IκBα–P65 axis is maintained in a continuously activated state during the pathogenesis of hepatic steatosis with high levels of systematic and local cytokine productions, which, combined with free fatty acids, can trigger the activation of JNK1/2 MAPK cascade, leading to the subsequent insulin dysfunction and abnormal fatty acid accumulation in the liver^24^,^51^,^52^. It has been reported that a transnormal upregualtion of TAK1–JNK/NF-κB in hepatocytes contributes to impaired hepatocytes homeostasis and expanded hepatic inflammation and cell death during liver injury^53^,^54^. In this study, the upregulation of TAK1 and NF-κB pathways during HFD-induced hepatic steatosis was greatly inhibited by liver-specific TRAF3 deficiency, suggesting the participation of TAK1–IKKβ–IκBα–P65 in TRAF3-mediated inflammation, insulin resistance and hepatic steatosis; however, the alternative NF-κB pathway was not significantly influenced by TRAF3 on HFD stimulation *in vivo* or on palmitate administration *in vitro*, although the regulation of the NIK-P100-P52/RelB cascade by TRAF3 has been extensively studied^33^. Differences in cell types, extracellular stimuli and upstream/downstream factors might explain the diversity of TRAF3-related molecular events and the resultant cellular behaviours.

Importantly, in the present study, mice receiving the treatment of a specific TAK1 inhibitor significantly ameliorated insulin resistance, glucose intolerance, inflammatory response and hepatic fatty acid accumulation triggered by HFD in NTG and in TRAF3-LTG mice. Consistent with *in vivo* results, artificial TAK1 downregulation greatly reversed the modulation of TRAF3 in palmitate-stimulated insulin signalling dysfunction and lipid accumulation in primary hepatocytes. Thus, the activation of TAK1 is indispensable during TRAF3-regulated hepatic steatosis and related metabolic disorder. However, intriguingly, Inokuchi-Shimizu *et al*.^55^ recently reported that the liver-specific KO of TAK1 exacerbated hepatic steatosis via suppressing fatty acid oxidation. Reasons for this conflicting conclusion about the role of TAK1 knockdown and TAK1 ablation in hepatic steatosis not yet fully understood; but, it should be noted that TAK1 is an essential regulator of various molecular and cellular events under physiologic conditions, and that mice with a TAK1 deficiency lose their ability to regulate a diverse range of cellular responses, particularly in response to stimuli inducing innate and adaptive immunity, suggesting an indispensible role of TAK1 in physiological events^56^. Therefore, it is possible that TAK1 accounts for the functional homeostasis of the liver and that maintaining TAK1 expression within a limited range, but not completely ablating, is helpful to protect the liver against steatosis.

The TAK1-dependent mechanism during TRAF3-regulated insulin resistance, inflammation and hepatic steatosis raises another question: how does TRAF3 regulate TAK1 activation? It has been demonstrated that the K63-linked polyubiquitination of TAK1 at residue K158 always occurs on extracellular stimuli, which is followed by autophosphorylation at T187 and S192 (refs 57, 58, 59). Previous studies indicated that TRAF3 possesses a C-terminal TRAF functional domain that renders TRAF3 an adaptor characteristic, whereas the RING finger motif in the N-terminal domain of TRAF3 endows TRAF3 with ubiquitin ligases activity^37^. Here we report that TAK1 ubiquitination can be induced by palmitate administration in hepatocytes, and TRAF3 absence significantly nullified this endogenous ubiquitination of TAK1. Biological investigations demonstrated that TRAF3 activates TAK1 protein kinase activity via a direct binding to TAK1, then the RING finger of TRAF3 ubiquitinates TAK1, leading to TAK1 phosphorylation and activation. However, the interaction between endogenous TRAF3 and TAK1 protein in hepatocytes remains to be shown. Once the proposed interaction between TRAF3 and TAK1 has been disrupted, the regulatory function of TRAF3 on HFD-induced hepatic steatosis was almost completely neutralized. Furthermore, the catalytically inactivated TRAF3 (C68A, H70A) mutant, which lost its E3 ligase activity, failed to influence TAK1–JNK1/2/NF-κB pathway and insulin signalling. Consequently, the TRAF3 (C68A, H70A) mutant treatment had a negligible effect on abnormal fatty acid accumulation in hepatocytes on palmitate administration. Therefore, the TRAF3–TAK1 interaction and the subsequent TAK1 ubiquitination is required for and contributes to the exacerbation of hepatic steatosis and insulin resistance mediated by hepatocyte TRAF3.

In conclusion, the present study identified a novel effect of hepatocyte TRAF3 on hepatic steatosis that is dependent on the interaction of TRAF3 with TAK1, which promotes the ubiquitination and phosphorylation of TAK1 and enhances the activation of downstream JNK and NF-κB cascades, thereby promoting insulin resistance, gluconeogenesis, inflammatory response and lipid accumulation in the liver on an HFD stimulus. Reducing TRAF3 expression or disrupting the TRAF3–TAK1 interaction in the liver might be a promising therapeutic approach for NAFLD and related metabolic diseases.

## Methods

### Reagents

Antibodies against the following proteins were purchased from Cell Signaling Technology: TRAF3 (4729, 1:1,000 dilution); p-IRS1^ser307^ (2831, 1:1,000 dilution); IRS1 (2832, 1:1,000 dilution); p-AKT^Ser473^ (4060, 1:1,000 dilution); AKT (4691, 1:1,000 dilution); p-GSK3β (9322, 1:1,000 dilution); GSK3β (9315, 1:1,000 dilution); FOXO1 (2880, 1:1,000 dilution); PEPCK (6924, 1:1,000 dilution); IKKβ (2370, 1:1,000 dilution); p-IκBα (9246, 1:1,000 dilution); IκBα (4814, 1:1,000 dilution); p-P65 (3033, 1:1,000 dilution); P65 (4764, 1:1,000 dilution); MKK7 (4172, 1:1,000 dilution); p-JNK (4668, 1:1,000 dilution); JNK (9252, 1:1,000 dilution); p-C-JUN^ser73^ (9164, 1:1,000 dilution); p-C-JUN^ser63^ (2361, 1:1,000 dilution); p-TAK1 (9339, 1:1,000 dilution); TAK1 (4505, 1:1,000 dilution); and GAPDH (2118, 1:1,000 dilution). Antibody against p-IRS1^Tyr608^ (09–432, 1:1,000 dilution) was purchased from Merck Millipore. An antibody against p-IKKβ (ab5915, 1:1,000 dilution) was obtained from Abcam (Cambridge, MA, USA). An antibody against p-POXO1 (TA323072, 1:500 dilution) was obtained from ORIGENE. Antibodies against G6Pase (ARP44223-P050, 1:1,000 dilution) and p-MKK7 (OAAF05547, 1:1,000 dilution) were obtained from AVIVA SYSTEMS BIOLOGY. Antibodies against Myc (Roche, Basel, Switzerland; 11814150001, 1:1,000 dilution) and Flag (Sigma, St Louis, MO, USA; F3165, 1:1,000 dilution) were also used. The BCA protein assay kit came from Pierce (Rockford, IL, USA). We used peroxidase-conjugated secondary antibodies (Jackson ImmunoResearch Laboratories, at 1:10,000 dilution) for visualization. Fetal bovine serum was purchased from Gibco (Waltham, MA, USA). Cell culture reagents and all other reagents were purchased from Sigma.

### Animals and treatment

The targeting construct for generation of TRAF3^flox/flox^ was prepared using a 129/Sv genomic BAC clone (clone no. 386H13) harbouring the complete TRAF3 locus^60^. The construct comprised a 3.0-kb 5′ homology arm, a PGK-EM7-neo selection cassette for positive selection by a G418 cassette and a 1.6-kb 3′ homology arm. Two loxP sites flanked a sequence containing exon 3 and the PGK-EM7-neo cassette, whereas the PGK-EM7-neo cassette alone was additionally flanked by two Flp recognition target (FRT) sites. Linearized targeting vectors were electroporated into the V6.5 embryonic stem cell (ES cell) line, which was derived from C57BL/6 and 129S6SvEv F1 hybrid mice. Colonies resistant to G418 were picked and expanded for screening. Then the Flp recognition target (FRT) sites flanking the neo cassette was removed through Flp-mediated recombination. Next, two heterozygous ES cells clones that had undergone homologous recombination were injected into C57BL/6J blastocysts, and the resulting chimeric male mice were mated with C57BL/6J female mice to obtain homozygous TRAF3^flox/flox^ mice^61^. The primer pairs P1 and P2, P5 and P6 were used for the PCR-based screening of ES cells, and the primers P3 and P4 were used to genotype the mice (Supplementary Table 2). Mice with TRAF3-LKO were generated through interbreeding TRAF3^flox/flox^ mice (on a C57BL/6J background; TRAF3-flox) with Albumin-Cre mice (Jackson Laboratory, Bar Harbor, ME, USA). TRAF3-flox mice without the introduction of Cre recombinase were used as littermate controls. To generate TRAF3-LTG, a construct containing full-length TRAF3 cDNA was first inserted into the CAG-loxp-CAT-loxp cassette followed by microinjection into fertilized C57BL/6J (WT) embryos. TRAF3-LTG mice were bred by crossing the obtained mice with Albumin-Cre mice. The female *ob/ob* mice used in the present study were purchased from the Jackson Laboratory (stock no.: 000632). To generate shTRAF3, hairpin-forming oligonucleotides TRAF3-f, 5′- CCGGGCAGCTTTGAGATCGAGATTGCTCGAGCAATCTCGATCTCAAAGCTGCTTTTTG -3′ and TRAF3-r, 5′- AATTCAAAAAGCAGCTTTGAGATCGAGATTGCTCGAGCAATCTCGATCTCAAAGCTGC -3′ were synthesized, annealed and subcloned into the shuttle vector distal to the U6 promoter. Using a similar process, recombinant adenovirus was generated (AdshTRAF3). To specifically knockdown TRAF3 in hepatocytes for *in vivo* experiments, AdshTRAF3 (5 × 10^9^ plaque-forming units) was injected into female *ob/ob* mice via the jugular vein at the age of 8 weeks after NC administration. Mice injected with AdshRNA were used as controls. AdTRAF3 and AdTRAF3-M (adenovirus encoding mutant TRAF3 with a TAK1-binding domain deletion) were generated and injected into C57BL/6J mice to overexpress TRAF3 or TRAF3-M in the liver. AdGFP was injected into mice as the corresponding control. The specific TAK1 inhibitor 5Z-7-oxozeaenol (5Z-7-ox; O9890-1 MG; Sigma) was administered intraperitoneally to NTG and TRAF3-LTG mice (5 mg kg^−1^) in parallel to inhibit TAK1 activation.

Male mice of 8–10 weeks of age (24–27 g) were used in these experiments. The mice were bred in a standard environment with a 12-h light/dark cycle. The hepatic steatosis model was established in mice through feeding an HFD (protein, 18.1%; fat, 61.6%; carbohydrates, 20.3%; D12492, Research Diets, New Brunswick, NJ, USA) continuously for 24 weeks. Mice administered an NC diet (protein, 18.3%; fat, 10.2%; carbohydrates, 71.5%; D12450B, Research Diets) served as controls. Food and water were provided *ad libitum*. The body weight and FBG level of mice were examined every 4 weeks. All animal protocols were approved by the Animal Care and Use Committee of Renmin Hospital at Wuhan University. The animals received humane care according to the criteria outlined in the ‘Guide for the Care and Use of Laboratory Animals' prepared by the National Academy of Sciences and published by the National Institutes of Health.

### Metabolic assays and serum cytokine analyses

FBG and FINS levels were examined after mice had fasted for 6 h by using a glucometer (One Touch Ultra Easy, Life Scan, Wayne, PA, USA) and by ELISA (Millipore, Billerica, MA, USA), respectively, and the HOMA-IR was calculated with the equation (FBG (mmol l^−1^) × FINS(mIU l^−1^))/22.5. To perform the glucose tolerance tests, 1 g kg^−1^ glucose (Sigma-Aldrich, St Louis, MO, USA) was i.p. injected into mice, whereas 0.75 U kg^−1^ insulin (Novolin R, Novo Nordisk, Bagsvaerd, Denmark) was i.p. injected into mice for insulin tolerance tests. Blood glucose levels were examined at 0, 15, 30, 60 and 120 min after injection. The concentrations of cytokines (TNF-α, IL-1β, IL-2, IL-4, IL-6, IL-10 and monocyte chemotactic protein-1) in the serum were examined by ELISA (R&D; MBL; RayBio; Invitrogen, Carlsbad, CA, USA; Peprotech). Commercial kits were employed to measure the TG, TC and NEFA content in the liver (290-63701 for TG assay, 294-65801 for TC assay, 294-63601 for NEFA assay; Wako, Tokyo, Japan) according to the manufacturer's instructions.

### Histological analyses

The morphological characteristics of livers were evaluated on liver sections prepared using the Tissue-Tek OCT compound (Sakura Finetek, Tokyo, Japan) or paraffin^62^. The frozen liver sections were subjected to oil red O staining to visualize lipid droplets in the liver, whereas haematoxylin and eosin staining was performed on paraffin sections after deparaffinization and rehydration to observe the distribution of lipid accumulation. Digital images were obtained with a light microscope (Olympus, Tokyo, Japan). For periodic acid–Schiff staining, the livers were collected and fixed in absolute ethyl alcohol for 36 h and then embedded in paraffin. After being deparaffinized and hydrated in deionized water, 5-μm liver sections were incubated in 0.5% periodic acid solution for 5 min at room temperature. Thereafter, the sections were washed and bathed with Schiff's reagent for 15 min at room temperature. After the sections were washed, they were counterstained with haematoxylin. The sections were then washed, dehydrated, cleared and mounted in xylene-based mounting media.

### Total glycogen assay

For the glycogen content assay, 100 mg of liver tissue was homogenized in 300 ml of isopropyl alcohol, after which centrifuged supernatants were collected. Total glycogen in the liver was measured according to the methods of the Glycogen Assay Kit (BioVision). The amount of glycogen was normalized to the amount of protein.

### Immunofluorescence

TRAF3 expression and localization in the liver sections of mice were investigated using TRAF3 and HNF4 (marker of hepatocytes) co-staining^63^. The mouse HNF4-specific antibody (ab41898, Abcam) and the rabbit anti-mouse TRAF3 antibody (ab76147; Abcam) were used as primary antibodies, and goat anti-mouse IgG and anti-rabbit IgG (Invitrogen) antibodies were applied as the corresponding secondary antibodies. Immunofluorescence images were obtained using fluorescence microscope (Olympus) running DP2-BSW software (version 2.2).

### Liver function assay

The serum levels of ALT, AST and ALP were examined using an ADVIA 2400 Chemistry System analyser (Siemens, Tarrytown, NY, USA) to evaluate the liver function of mice in the indicated groups, according to the manufacturer's instructions.

### Human liver samples

NAFLD liver samples were obtained from patients with hepatic steatosis who had undergone liver biopsy or transplantation. Control liver samples were collected from donors whose livers were unsuitable for transplantation for non-hepatic reasons. Written informed consent was obtained from subjects or families of liver donors. Clinical and histologic characteristics of these samples were provided in the Supplementary Tables 3 and 4. All procedures that involved human samples were approved by the Renmin Hospital of Wuhan University Review Board, Wuhan, China, and were consistent with the principles outlined in the Declaration of Helsinki.

### Primary hepatocyte isolation and culture

Primary hepatocytes from 6- to 8-week-old mice were isolated by liver perfusion. Briefly, mice were anesthetized with pentobarbital sodium (90 mg kg^−1^). A total of 45 ml of Liver Perfusion Medium (Life Technologies, Carlsbad, CA, USA, 17701–038) was perfused through the portal vein, followed by the perfusion of Liver Digest Medium (Life Technologies, 17703-034) for 5 min at a rate of 2 ml min^−1^. After this digestion, the liver was excised, minced and filtered through a steel mesh (100 μm, Life Technologies). Primary hepatocytes were separated via centrifugation at 50*g* for 5 min and purified on 50% Percoll solution (17-0891-01, GE Healthcare Life Sciences, Buckinghamshire, England). The obtained hepatocytes were resuspended in Dulbecco's modified Eagle's medium. Trypan blue exclusion assays indicated that cell viability was >95%. Hepatocytes were cultured in Dulbecco's modified Eagle's medium supplemented with 10% fetal bovine serum and 1% penicillin–streptomycin in a 5% CO_2_/water-saturated incubator at 37 °C. Palmitate (0.25 mM; Sigma-Aldrich) was added to the medium for 24 h to establish the *in vitro* model of lipid accumulation in hepatocytes.

### Cell lines

All cell lines used in this study, including the normal human hepatocyte cell line L-02, HepG2 and 293T cells were purchased from the Type Culture Collection of the Chinese Academy of Sciences, Shanghai, China. Mycoplasma contamination was checked at least once in a month. None of the cell lines were authenticated in house by short tandem repeat DNA profiling.

### *In vitro* HGP assays

HGP was measured in primary hepatocytes as follows: primary hepatocytes were incubated for 4 h at 37 °C in Kreb–Ringer bicarbonate buffer (119 mM NaCl, 5 mM KCI, 2.6 mM KH_2_PO_4_, 2.6 mM MgSO_4_, 2 mM CaCl_2_, 24.6 mM NaHCO_3_ and 10 mM HEPES, pH 7.4) supplemented with 0.5% BSA and gluconeogenic substrates (10 mM lactate and 5 mM pyruvate) in the presence or absence of glucagon. The glucose concentrations in the culture medium were measured using Glucose LiquiColor Kits (Fisher Scientific Inc.). Glucose production was normalized to the total hepatocyte protein concentrations^32^,^64^.

### Intracellular TG level

Twenty-four hours after treatment with palmitate, cultured L-O2 cell lines were collected by centrifugation at 1,000*g* for 10 min at 4 °C. The intracellular TG levels were measured using a commercially available TG Colorimetric Assay Kit (Cayman, Ann Arbor, MI) according to the manufacturer's protocol.

### Quantitative RT–PCR and western blot analysis

For the RT–PCR assay, total mRNA was isolated from liver samples or cultured cells using TRIzol Reagent (15596-026, Invitrogen) and reverse-transcribed into cDNA using a Transcriptor First Strand cDNA Synthesis Kit (04896866001, Roche) according to the manufacturer's protocol. SYBR Green (04887352001, Roche) was applied to quantify PCR amplification. The mRNA expression levels of target genes were normalized to GAPDH expression^65^. The primer pairs used in this study are listed in Supplementary Table 5.

For western blot analyses, total protein samples were isolated from tissue or cell samples using RIPA lysis buffer (720 μl of RIPA, 20 μl of phenylmethyl sulphonyl fluoride, 100 μl of complete protease inhibitor cocktail, 100 μl of Phos-stop, 50 μl of NaF and 10 μl of Na_3_VO_4_ in a final volume of 1 ml), and the protein concentrations were examined using a Pierce BCA Protein Assay Kit (23225, Thermo Fisher Scientific, Rockford, IL, USA). A total of 50 μg obtained protein samples was separated in a 10% SDS–PAGE gel (NP0301BOX, Invitrogen) and then transferred to a polyvinylidene difluoride membrane (IPVH00010, Millipore Corporation). After being blocked with 5% skim milk, the polyvinylidene difluoride membrane was incubated with primary antibodies at 4 °C overnight, followed by the corresponding secondary antibodies^66^. A ChemiDoc MP Imaging System (Bio-Rad, Hercules, CA, USA) was used for signal detection. Full gel scans for entire blots were provided in Supplementary Fig. 13. Protein expression levels were quantified with Image Lab Software and normalized to the loading control GAPDH.

### Plasmid constructs

The human *TRAF3* gene was cloned into psi-EGFP-myc-C1 to generate psi-EGFP-Myc-TRAF3. Human cDNA was PCR-amplified using the primers TAK1-1F and TAK1-579R, digested with BamHI and XhoI, and ligated into pcDNA5-Flag to create pcDNA5-Flag-TAK1. Consequently, the psi-EGFP-Myc-TRAF3, pcDNA5-Flag-TAK1 and DNA constructs corresponding to TRAF3 fragments (residues 1–266, 1–376, 267–568 and 267–376) and TAK1 fragments (residues 1–300, 1–480, 301–579 and 481–579) were constructed. The products of TRAF3 and TAK1 residues were digested with BamHI and XhoI and ligated into psi-EGFP-Myc-C1 and psi-Flag-cherry-C1, respectively. Primers for the creation of these constructs are provided in Supplementary Table 6.

### Immunoprecipitation

Cultured HepG2 cells were transiently transfected with EGFP-Myc-TRAF3 and Flag-TAK1 using FuGENE transfection reagent (Roche) according to the manufacturer's instructions and were incubated for an additional 48 h. Then, the cells were lysed in an ice-cold immunoprecipitation buffer containing a protease inhibitor cocktail, followed by centrifugation at 13,000*g* for 15 min. The obtained cell lysates were precleared with Protein A/G-agarose beads (11719394001 and 11719386001, Roche) for 3 h and then incubated with the indicated antibody at 4 °C overnight. The immunocomplex was collected after washing with cold immunoprecipitation buffer and subjected to immunoblotting using the indicated primary antibodies and the corresponding secondary antibodies.

### *In vivo* ubiquitination assays

The *in vivo* ubiquitination assay was performed as follows: briefly, cells were lysed in SDS lysis buffer (20 mM Tris-HCl, pH 7.4, 150 mM NaCl, 1 mM EDTA and 1% SDS) containing Protease Inhibitor Cocktail Tablets (Roche), and denatured by heating for 5 min. The supernatants were diluted 10-fold with lysis buffer (20 mM Tris-HCl, pH 7.4, 150 mM NaCl, 1 mM EDTA and 1% Triton X-100) containing Protease Inhibitor Cocktail Tablets (Roche). After centrifugation at 20,000 r.p.m. for 30 min at 4 °C, the supernatants were subjected to immunoprecipitation with the indicated antibodies^67^.

### *In vitro* ubiquitination assays

The tested proteins were expressed with a TNT Quick Coupled Transcription/Translation Systems Kit (Promega) following the manufacturer's instructions. Ubiquitination was analysed with a ubiquitination kit (Enzo Life Sciences) following the manufacturer's instructions.

### Statistical analysis

Appropriate statistical analyses were applied, assuming a normal sample distribution, as specified in the figure legends. All data in this study are expressed as the mean±s.d. Student's two-tailed *t*-test was used to compare the means of two groups of samples, and two-way analysis of variance with general linear model procedures using a univariate approach was applied for more than two groups. All statistical analyses were performed with SPSS software, version 13.0. A *P* value <0.05 was considered significant. No statistical method was used to predetermine sample size. No data were excluded. Randomization and blinding strategy was used whenever possible. Animal cohort sizes were determined on the basis of similar previous studies.

## Additional information

**How to cite this article:** Wang, P.-X. *et al*. Hepatocyte TRAF3 promotes liver steatosis and systemic insulin resistance through targeting TAK1-dependent signalling. *Nat. Commun.* 7:10592 doi: 10.1038/ncomms10592 (2016).

## Supplementary Material

Supplementary Information Supplementary Figures 1-13 and Supplementary Tables 1-6.
