# Supplementary material for: Hepatocyte TRAF3 promotes liver steatosis and systemic insulin resistance through targeting TAK1-dependent signalling
Source: Nat Commun. 2016 Feb 17;7:10592. doi: 10.1038/ncomms10592 (PMC4757796; doi:10.1038/ncomms10592)
Supplement: Supplementary Information — Supplementary Figures 1-13 and Supplementary Tables 1-6. [file ncomms10592-s1.pdf]

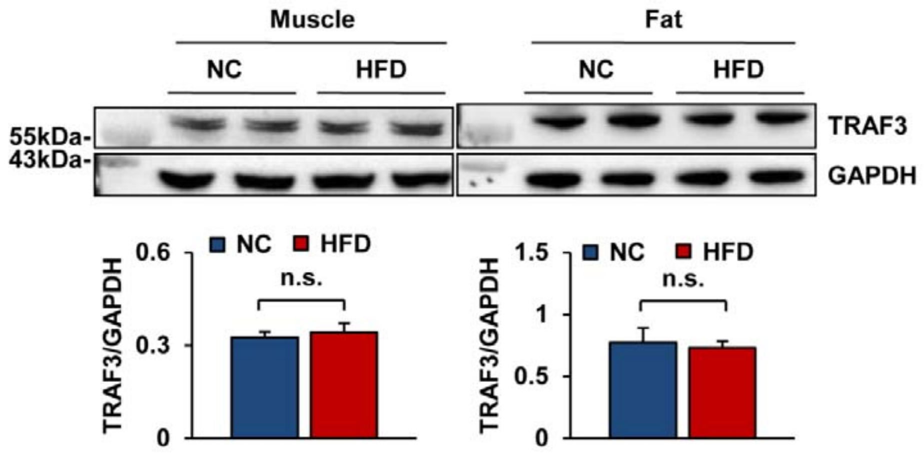

Supplementary Figure 1. TRAF3 protein expression in the muscle and fat samples from mice treated with NC or HFD for 24 weeks. n=4 for each group. n.s. no significant difference. The data represent as the mean ± s.d. Statistical analysis was carried out by Student's two-tailed t-test.

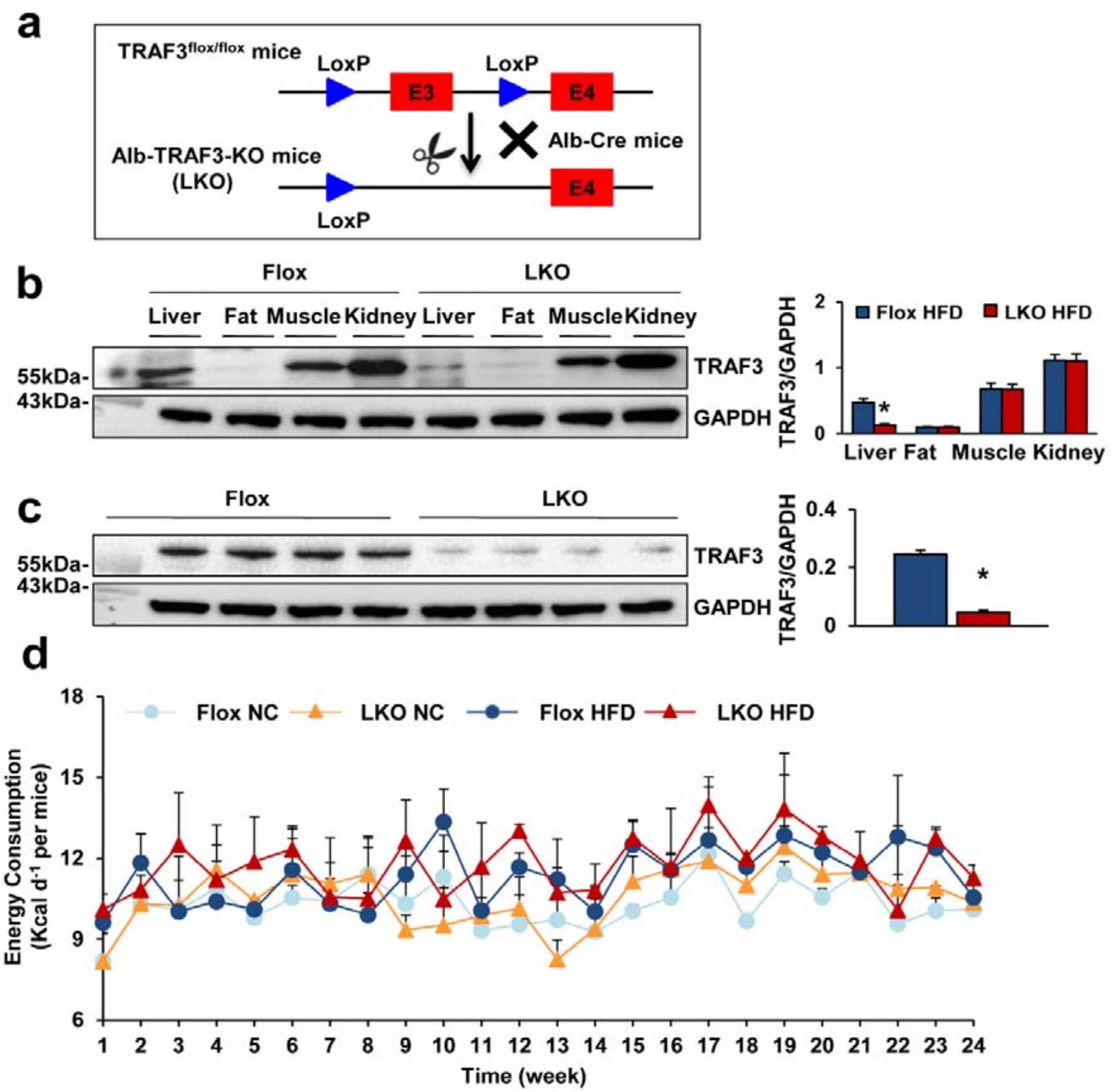

Supplementary Figure 2. TRAF3 specific deficiency in the liver fails to change energy consumption upon HFD treatment. (a) Schematic image of the generation of liver-specific TRAF3-KO mouse (TRAF3-LKO) strain. **(b)** Identification of the specific knockout of TRAF3 in the liver of TRAF3-LKO mice (n=4 for each group). *P<0.05 vs. the corresponding TRAF3-flox controls. **(c)** TRAF3 expression in the liver samples of TRAF3-LKO and TRAF3-flox mice were examined by western blot analysis (n=4). *P<0.05 vs. TRAF3-flox group. **(d)** Energy consumption calculated from food intake of mice in TRAF3-flox and TRAF3-LKO groups treated with HFD or NC control diet from 1-24 week. All values are means ± s.d. Significance determined by Student's two-tailed t-test **(b and c)** and two-way analysis of variance with general linear model procedures using a univariate approach **(d)**.

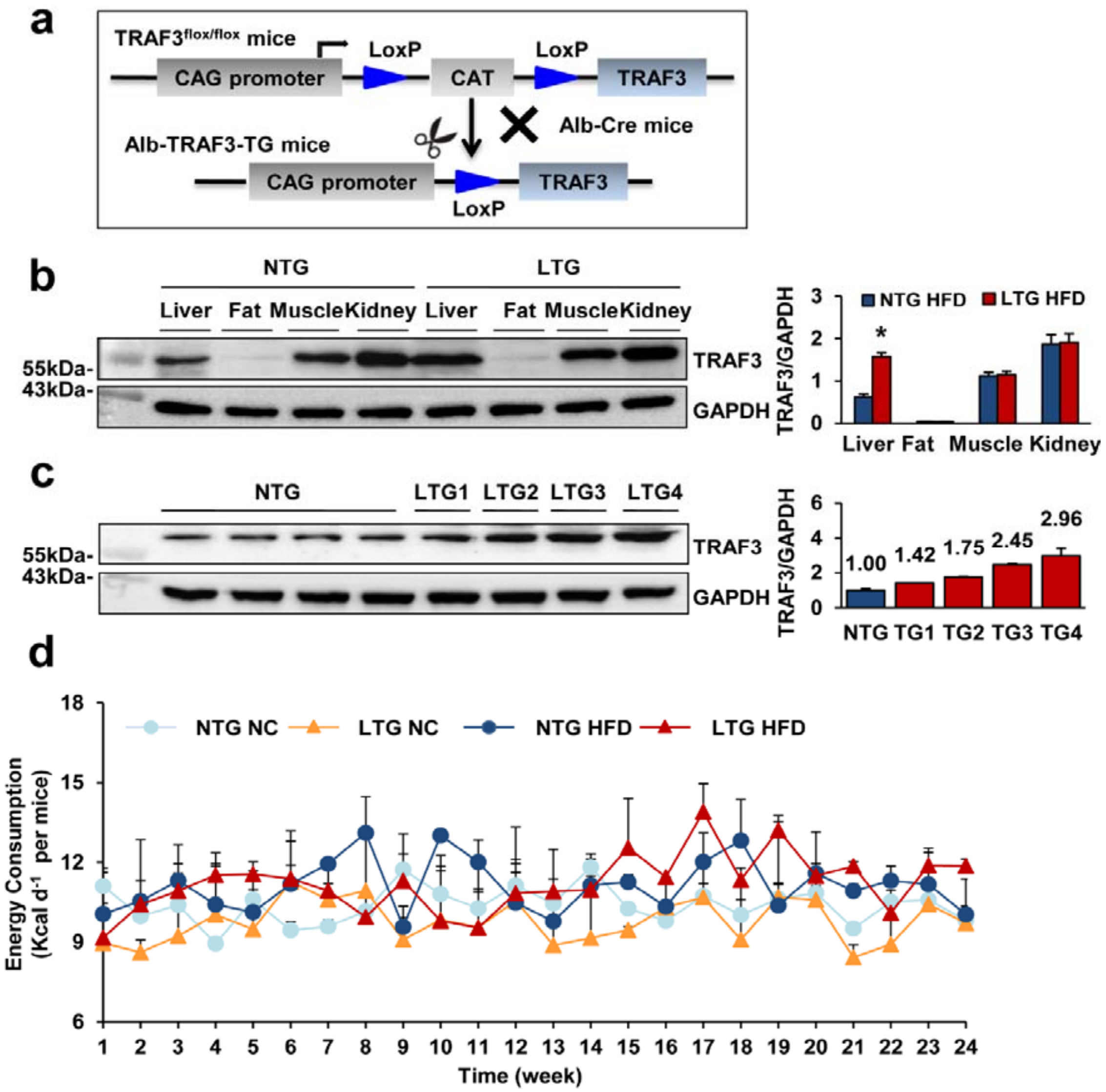

Supplementary Figure 3. Hepatic TRAF3 overexpression does not significantly influence energy consumption upon HFD administration. (a) Schematic workflow of the establishment of the liver-specific TRAF3-TG (TRAF3-LTG) mouse strains. **(b)** TRAF3 protein expression in the livers and other organs of mice with TRAF3-LTG and NTG controls (n=4). **(c)** Western blotting reveals TRAF3 expression in the liver samples of different TRAF3-LTG mouse lines (n=4 for each line). *P<0.05 vs. NTG controls. **(d)** Energy consumption of mice in NTG/NC, TRAF3-LTG/NC, NTG/HFD, and TRAF3-LTG/HFD groups during 1-24 week. All values are means ± s.d. Significance determined by Student's two-tailed t-test **(b)** and two-way analysis of variance with general linear model procedures using a univariate approach **(d)**.

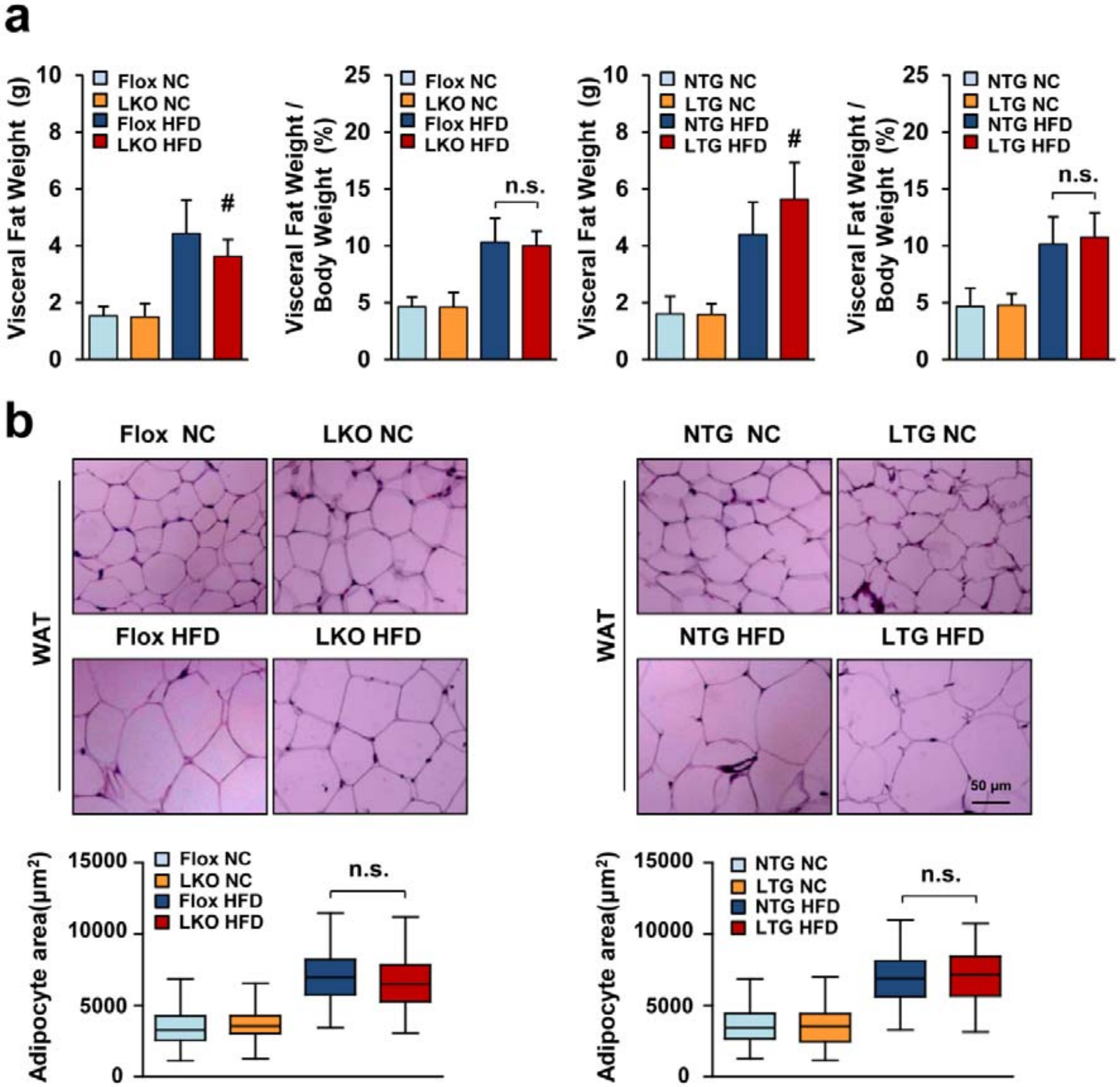

Supplementary Figure 4. TRAF3 promotes weight gain of adipose tissue without changing adipocytes enlargement induced by HFD. (a) Visceral fat weights of TRAF3-LKO, TRAF3-LTG, and their littermate controls after a 24-week of HFD or NC treatment. n=16-25/pre group. **(b)** The representative H&E staining images (upper panel) and the analyzed adipocyte areas (bottom column plots) on the white adipose tissue (WAT) sections of mice in the indicated groups. n=4-6 for each group. # $P<0.05$ vs. TRAF3-flox/HFD or NTG/HFD group. n.s., no significant difference. The data represent as the mean ± s.d. Significance determined by two-way analysis of variance with general linear model procedures using a univariate approach.

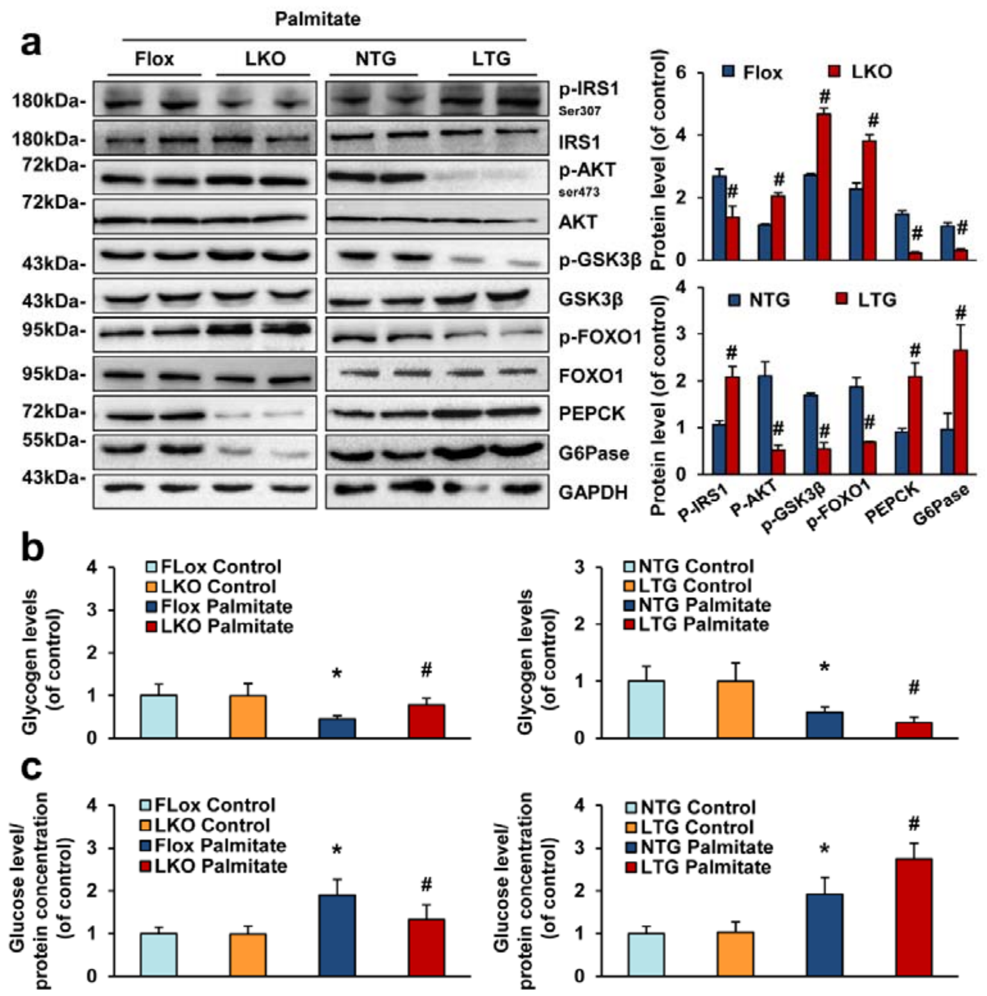

Supplementary Figure 5. TRAF3 regulates insulin signaling, glycogen level, and glucose content in hepatocytes upon palmitate administration *in vitro*. (a) The expression levels of key proteins in insulin signaling and glucogenesis-related cascades in palmitate-treated primary hepatocytes isolated from mice in TRAF3-LKO, TRAF3-LTG and their corresponding control groups. The expression of GAPDH or indicated total protein is loading control. **(b)** Glycogen levels in primary TRAF3-flox, TRAF3-LKO, NTG, and TRAF3-LTG hepatocytes upon palmitate stimulation for 24 h. n=3 independent experiments. **(c)** Ratios of glucose content to protein concentration in primary hepatocytes in the indicated groups after palmitate administration for 24 h.*P<0.05 vs. TRAF3-flox/control or NTG/control group; #P<0.05 vs. TRAF3-flox/palmitate or NTG/palmitate group. All values are means ± s.d. Significance determined by Student's two-tailed t-test **(a)** and two-way analysis of variance with general linear model procedures using a univariate approach **(b and c).**

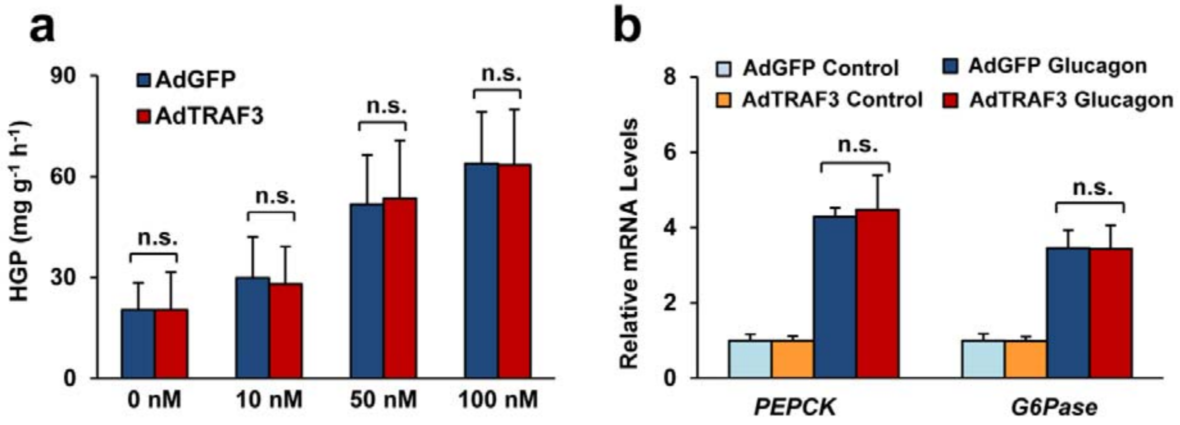

Supplementary Figure 6. TRAF3 shows no significant influence in hepatocyte glucose production induced by glucagon. Primary hepatocytes were infected with AdTRAF3 or AdGFP control followed by glucagon administration. Hepatocyte glucose production (HGP, **a**) and mRNA expression of PEPCK and G6Pase (**b**) were respectively measured in the medium and cells. n=3 independent experiments. n.s., no significant difference. All values are means ± s.d. Significance determined by Student's two-tailed t-test **(a)** and two-way analysis of variance with general linear model procedures using a univariate approach **(b).**

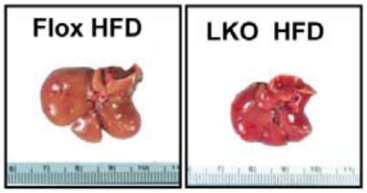

Supplementary Figure 7. Represent photographs of livers from TRAF3-flox and TRAF3-LKO mice administered with a HFD for 24 weeks.

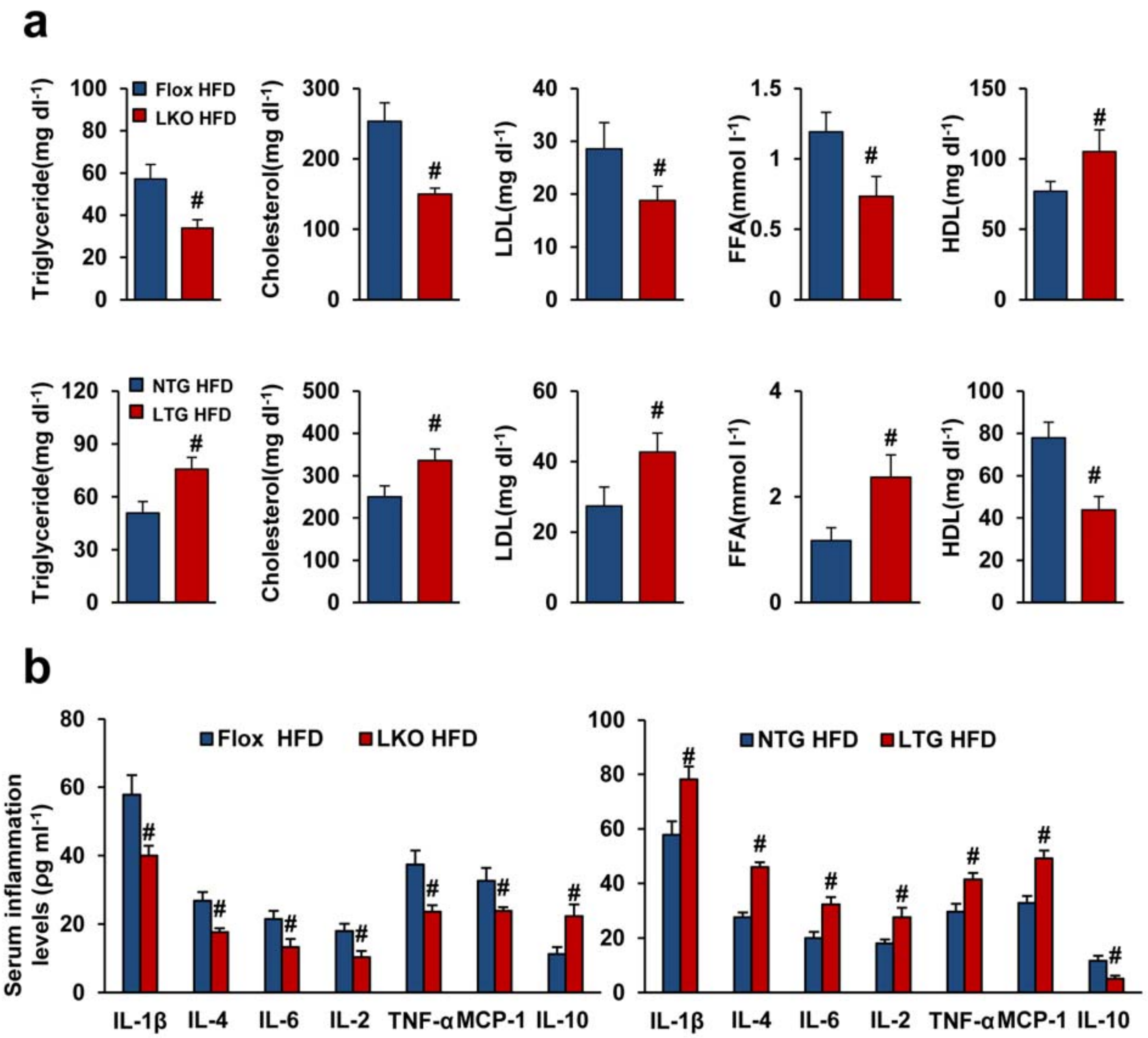

Supplementary Figure 8. TRAF3 exacerbates circulating lipid content disorder and inflammatory response upon HFD treatment. (a) The different types of fatty acids in the serum of mice in TRAF3-flox and TRAF3-LKO as well as in NTG and TRAF3-LTG groups after a 24-week HFD administration. **(b)** The serum cytokines and chemokines levels in mice of indicated groups after HFD treatment for 24 weeks. n=6-8 for each group. #*P*<0.05 vs. TRAF3-flox/HFD or NTG/HFD group. All values are means ± s.d. Significance determined by Student's two-tailed t-test.

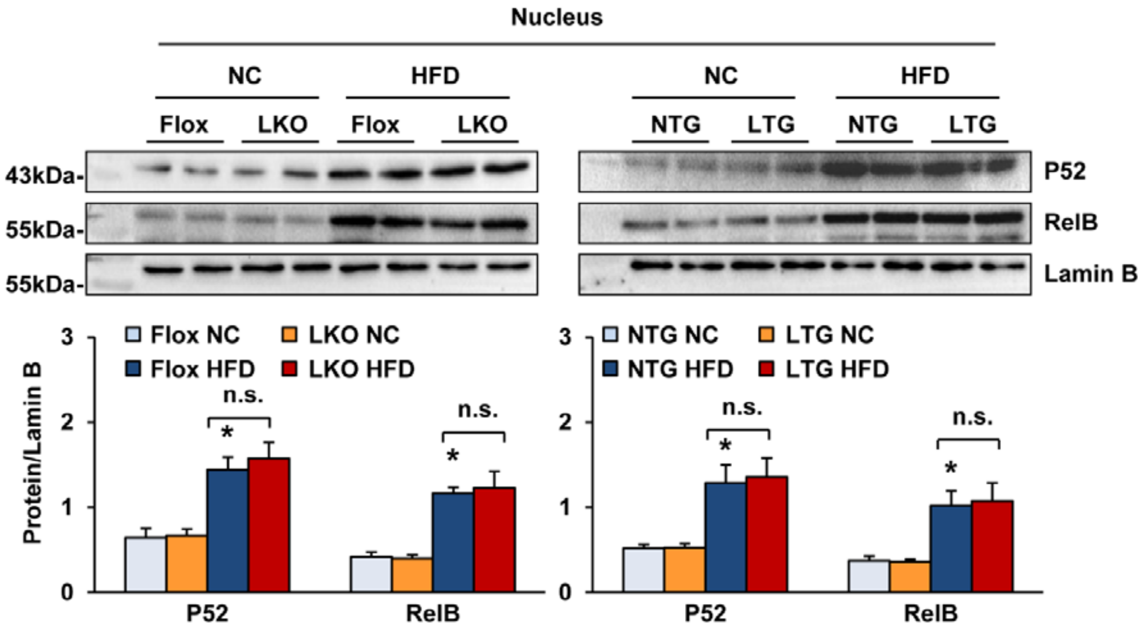

Supplementary Figure 9. Protein expression levels of P52 and RelB in the nucleus extract of liver samples from mice in TRAF3-flox, TRAF3-LKO , NTG, and TRAF3-LTG groups after NC or HFD treatment for 24 weeks. The expression level of lamin B serves as loading control. **P*<0.05 vs. TRAF3-flox/NC or NTG/NC group. n.s., no significant difference. All values are means ± s.d. Significance determined by two-way analysis of variance with general linear model procedures using a univariate approach.

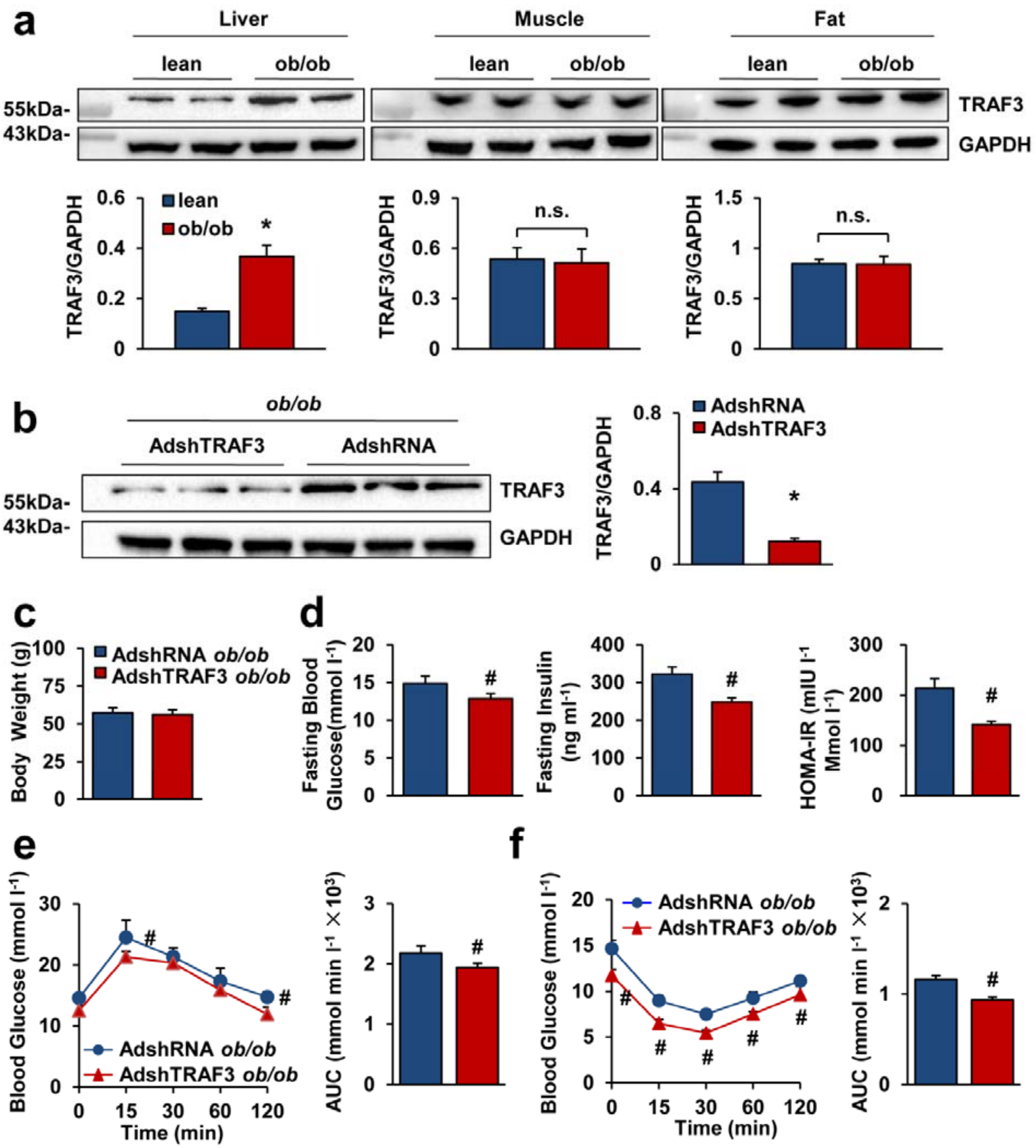

a
Liver
Muscle
Fat
lean
ob/ob
55kDa-
43kDa-
TRAF3
GAPDH
TRAF3/GAPDH
lean
ob/ob
*
n.s.
b
ob/ob
AdshTRAF3
AdshRNA
AdshRNA
AdshTRAF3
c
Body Weight (g)
AdshRNA ob/ob
AdshTRAF3 ob/ob
d
Fasting Blood Glucose(mmol l-1)
Fasting Insulin (ng ml-1)
HOMA-IR (mIU l-1 Mmol l-1)
#
e
Blood Glucose (mmol l-1)
Time (min)
AUC (mmol min l-1 ×10³)
f

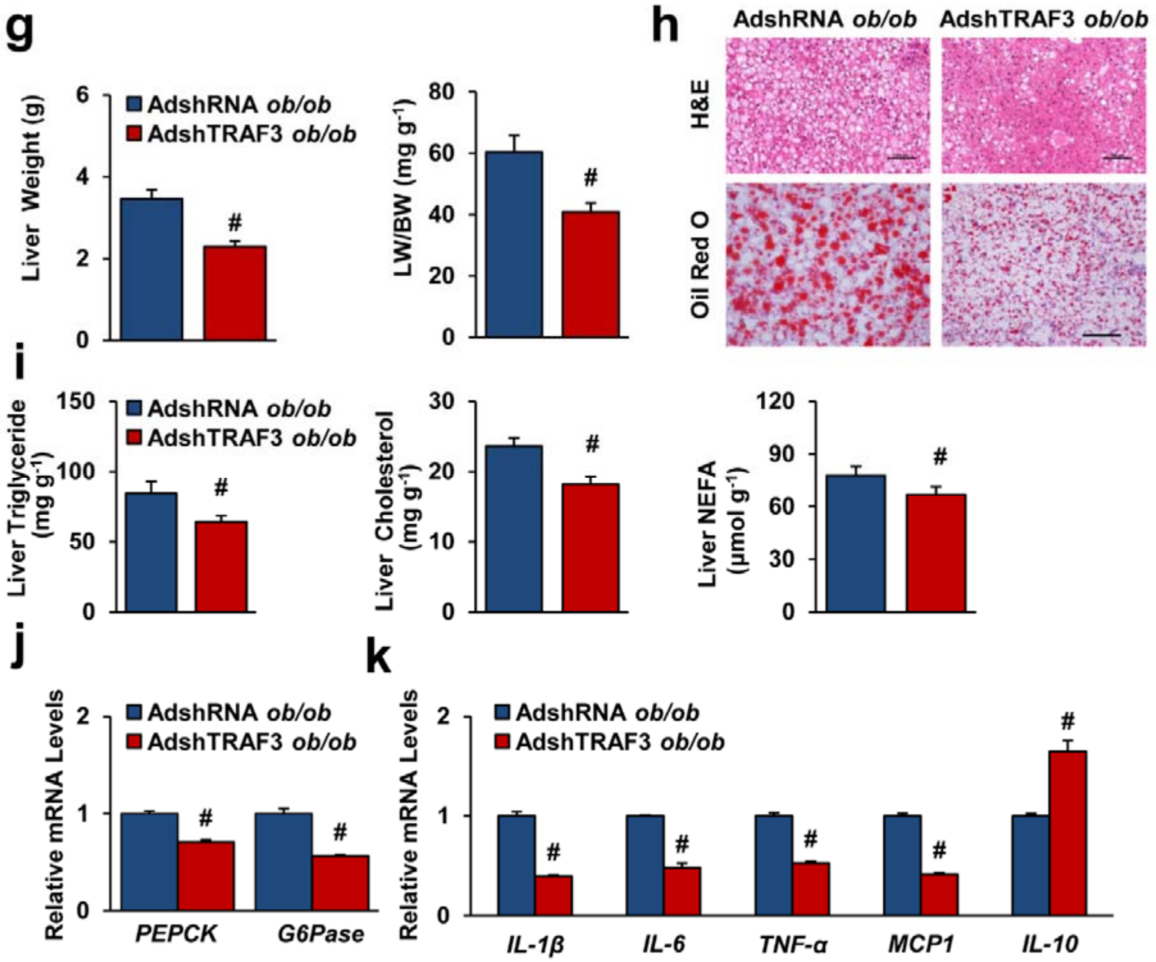
g
Liver Weight (g)
AdshRNA ob/ob
AdshTRAF3 ob/ob
LW/BW (mg g-1)
h
AdshRNA ob/ob
AdshTRAF3 ob/ob
H&E
Oil Red O
i
Liver Triglyceride (mg g-1)
Liver Cholesterol (mg g-1)
Liver NEFA (μmol g-1)
j
Relative mRNA Levels
PEPCK
G6Pase
k
IL-1β
IL-6
TNF-α
MCP1
IL-10

(continued)

Supplementary Figure 10. Liver-specific TRAF3 knockdown prevents against insulin resistance, hepatic steatosis and inflammatory response in *ob/ob* mice. (a) TRAF3 expression in the liver, muscle, and fat of *ob/ob* mice and lean controls fed with NC diet (n=4). $^*P<0.05$ compared with lean controls. n.s., no significant difference. **(b)** Identification of the downregulation of TRAF3 in the liver by injecting AdshTRAF3 in *ob/ob* mice. Mice injected with AdshRNA serve as controls. **(c)** The body weights of *ob/ob* mice after 4 weeks of administration of AdshTRAF3 or AdshRNA control (n=14–22). **(d)** Fasting blood glucose, fasting serum insulin, and the calculated HOMA-IR of *ob/ob* mice injected with AdshTRAF3 or AdshRNA at 8 week of age and fed for an additional 4 weeks (n=8–12). **(e and f)** Glucose and insulin tolerance of *ob/ob* mice was examined via IPGTT **(e)** and IPITT **(f)** assays, respectively, 4 weeks after AdshTRAF3 or AdshRNA injection. AUC of blood glucose levels was calculated (n=8–12 per group). **(g)** The liver weight and the LW/BW ratio of *ob/ob* mice after 4 weeks of AdshTRAF3 or AdshRNA treatment (n=14–22). **(h)** Represent H&E (upper) and oil red O (bottom) staining images of liver sections from *ob/ob* mice in AdshTRAF3 and AdshRNA groups (n=8–12). Scan bar=100 μm. **(i)** TG, TC and NEFA levels in the liver samples of *ob/ob* mice injected with AdshTRAF3 or AdshRNA controls (n=6–8). **(j and k)** mRNA expression of PEPCK and G6Pase **(j)** and inflammation-related genes **(k)** in the liver samples of AdshTRAF3 or AdshRNA-treated *ob/ob* mice (n=6-8). $\#P<0.05$ compared with AdshRNA-treated *ob/ob* mice. All values are means ± s.d. Significance determined by Student's two-tailed t-test.

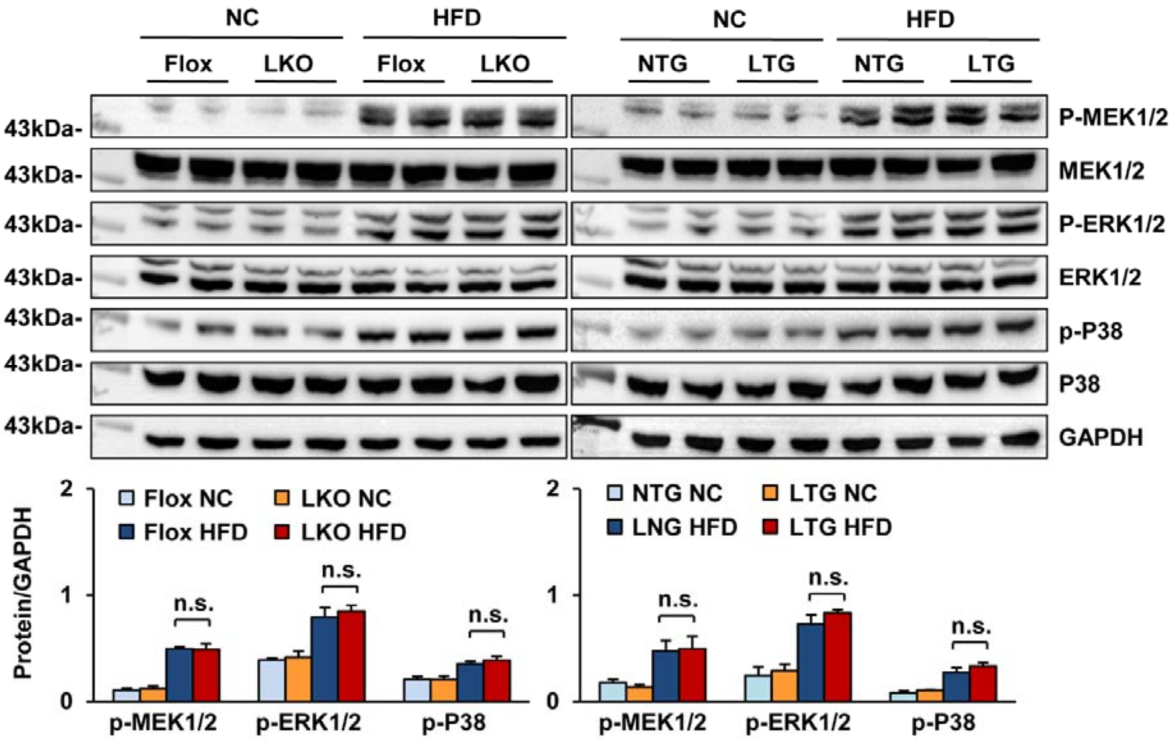

Supplementary Figure 11. The expression levels of P-MEK1/2, P-ERK, P-p38 and their corresponding total proteins in the liver samples of HFD- or NC-treated mice in TRAF3-flox, TRAF3-LKO , NTG, and TRAF3-LTG groups for 24 weeks (n=4). n.s., no significant difference. All values are means ± s.d. Significance determined by two-way analysis of variance with general linear model procedures using a univariate approach.

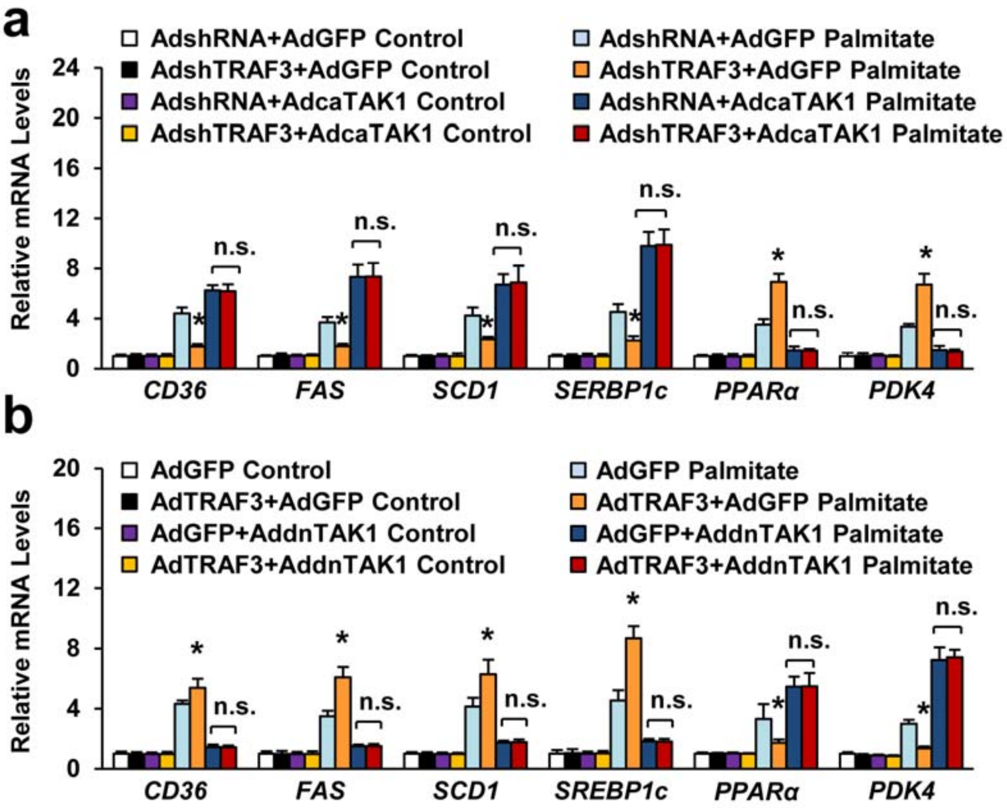

Supplementary Figure 12. mRNA expression of genes responsible for fatty acid metabolisms in LO2 cells co-infected with AdshTRAF3 and AdcaTAK1 (a), or with AdTRAF3 and AddnTAK1 (b) and stimulated with palmitate for 24 h. AdGFP or AdshRNA infected cells were served as controls. n=3 independent experiments. *P<0.05 vs. AdshRNA+AdGFP/palmitate or AdGFP/palmitate group. n.s., no significant difference. All values are means ± s.d. Significance determined by two-way analysis of variance with general linear model procedures using a univariate approach.

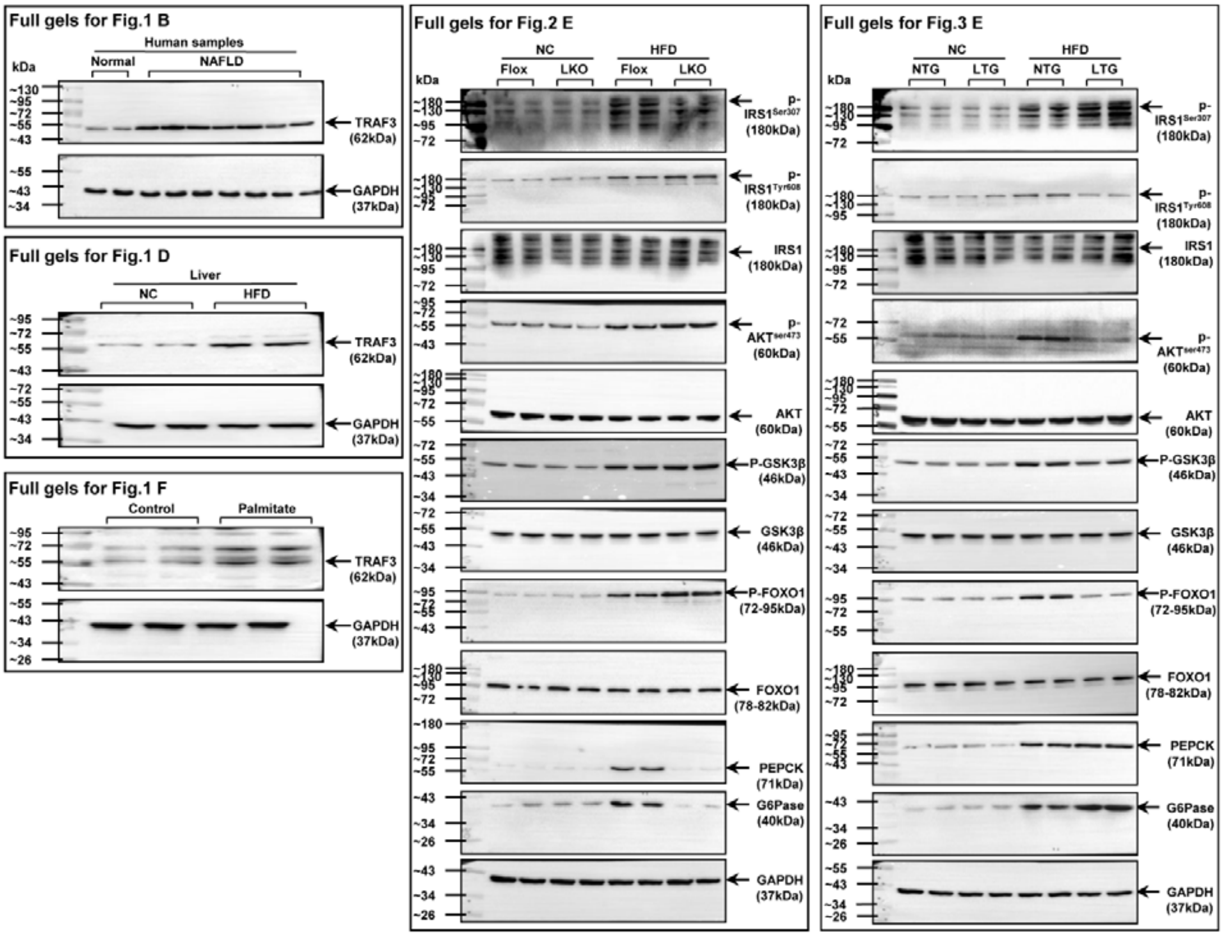

Supplementary Figure 13. Full gel scans relating to indicated figures.

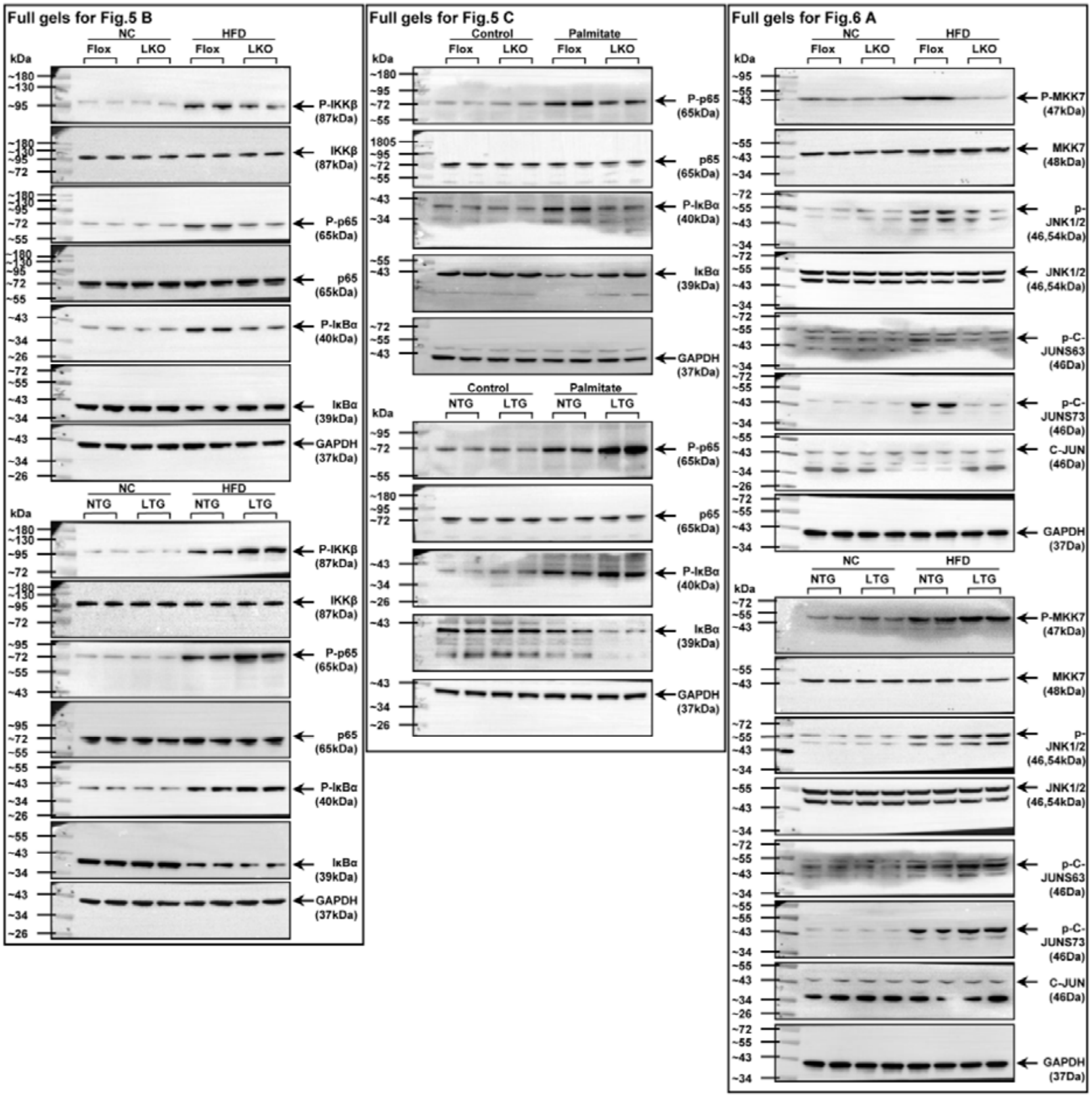

Supplementary Figure 13. Full gel scans relating to indicated figures (continued).

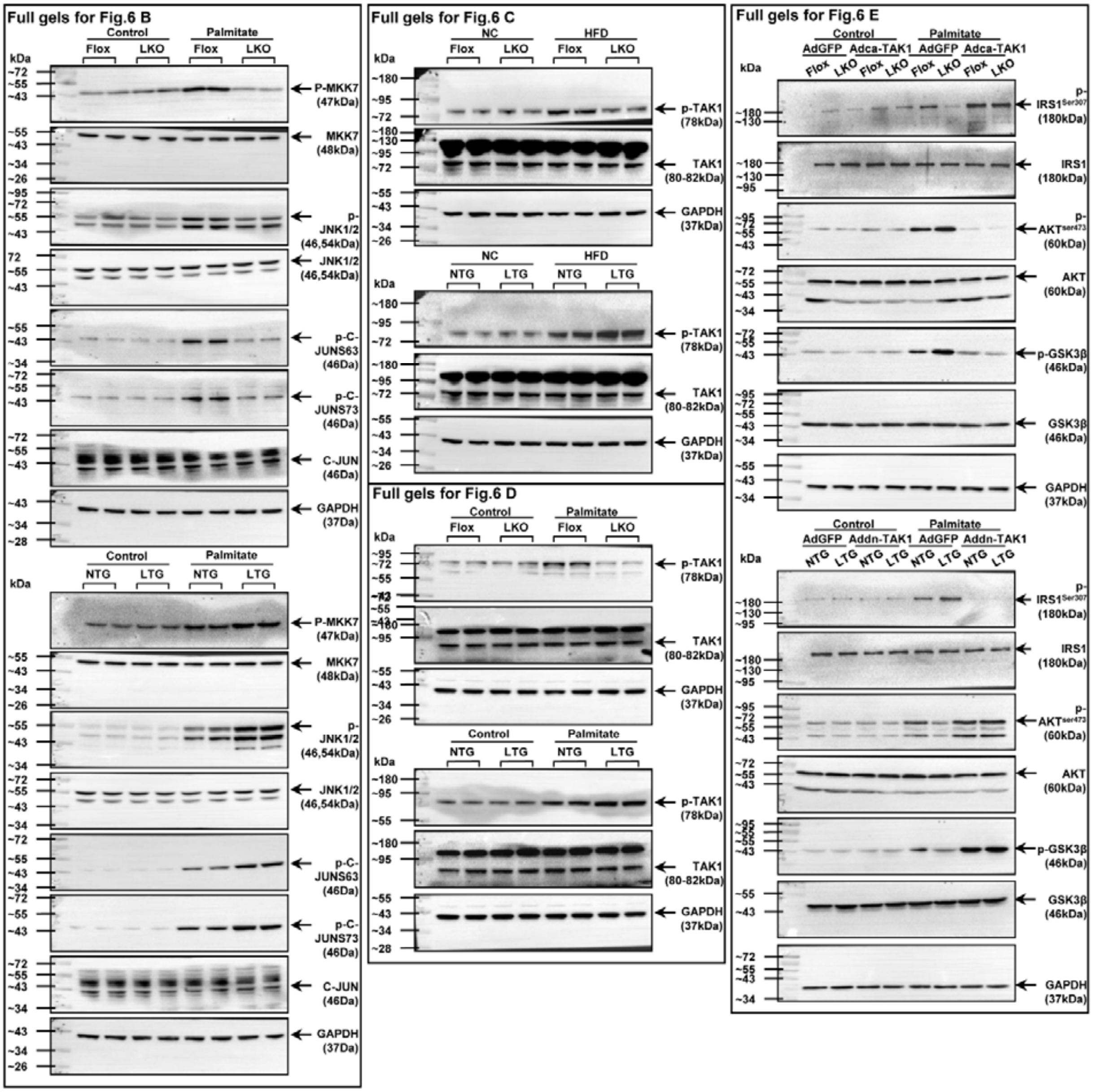

Supplementary Figure 13. Full gel scans relating to indicated figures (continued).

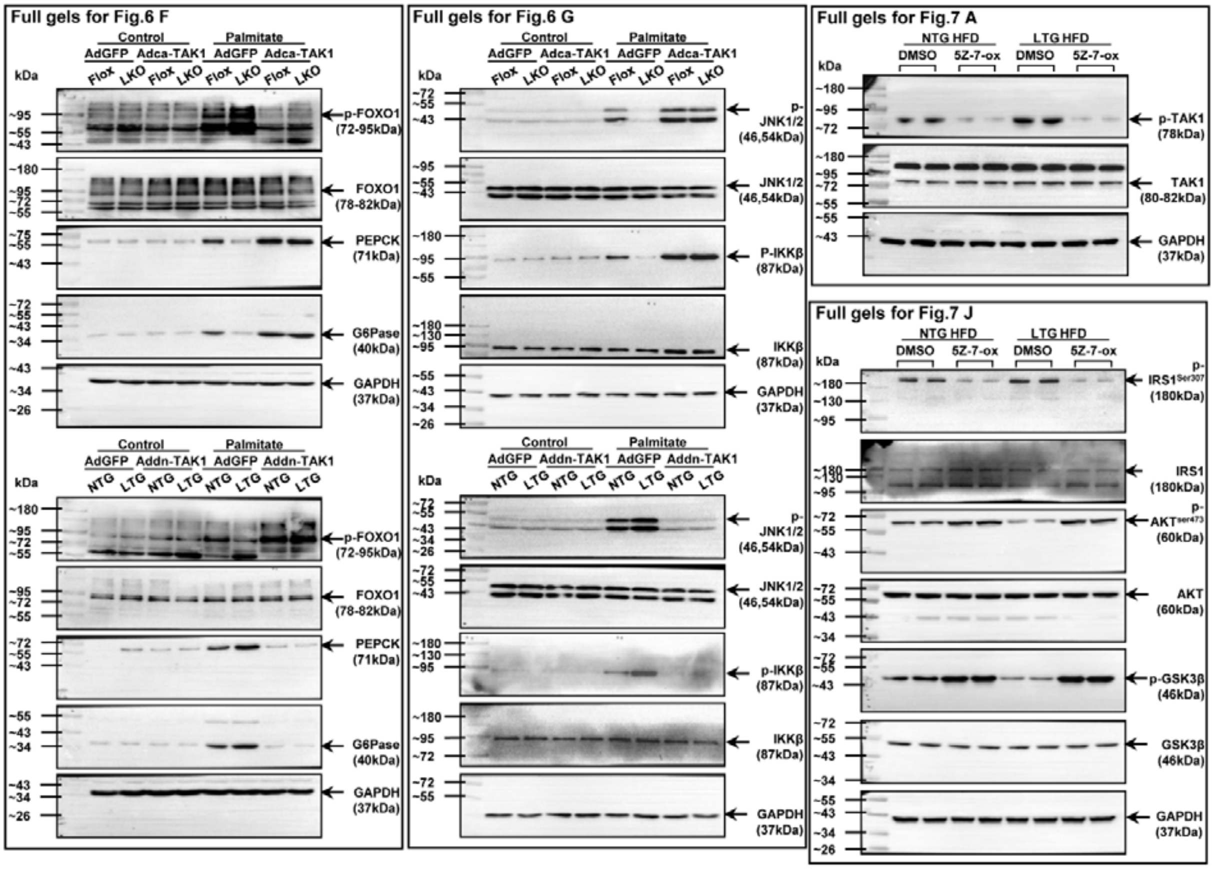

Supplementary Figure 13. Full gel scans relating to indicated figures (continued).

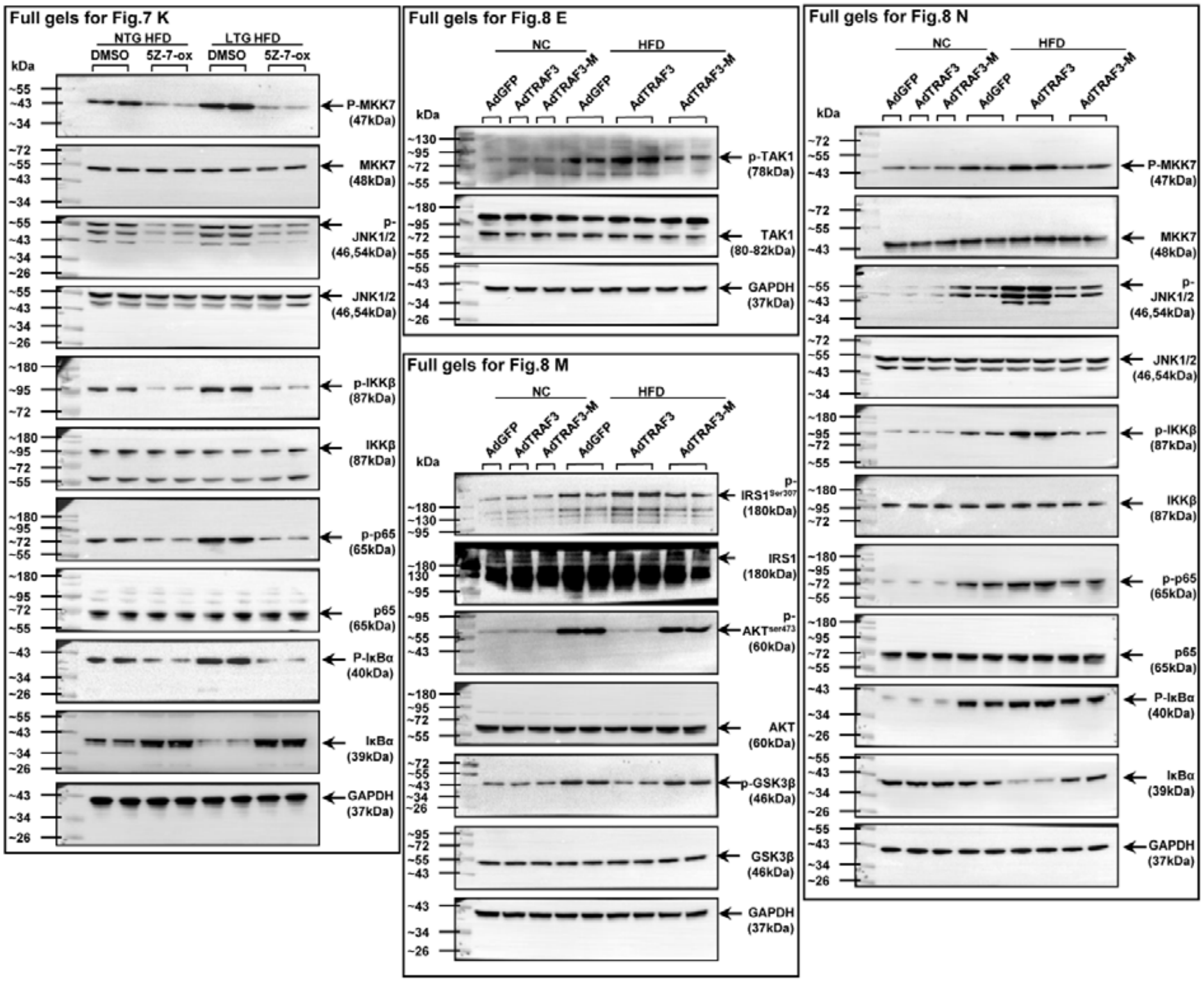

Supplementary Figure 13. Full gel scans relating to indicated figures (continued).

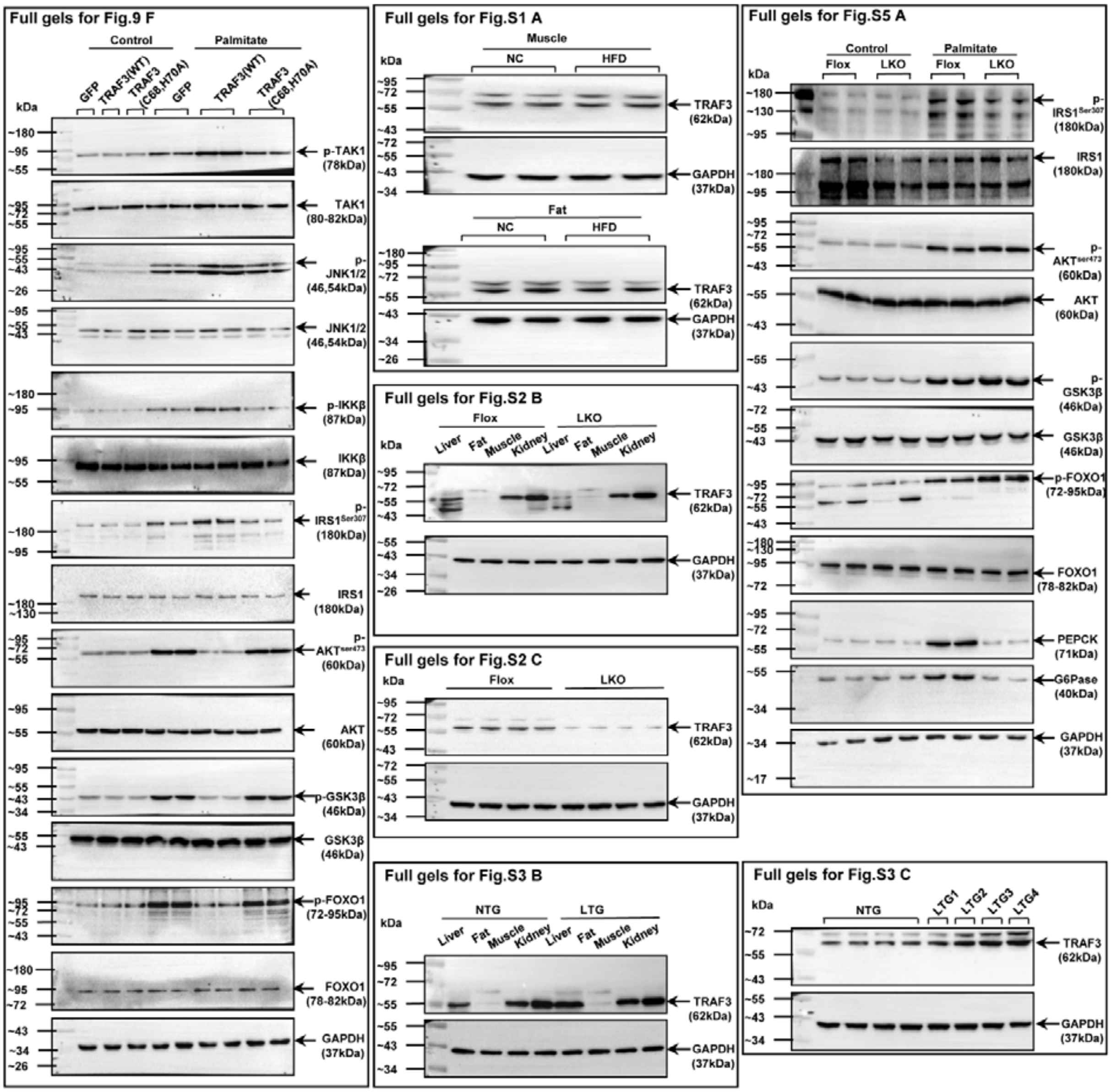

Supplementary Figure 13. Full gel scans relating to indicated figures (continued).

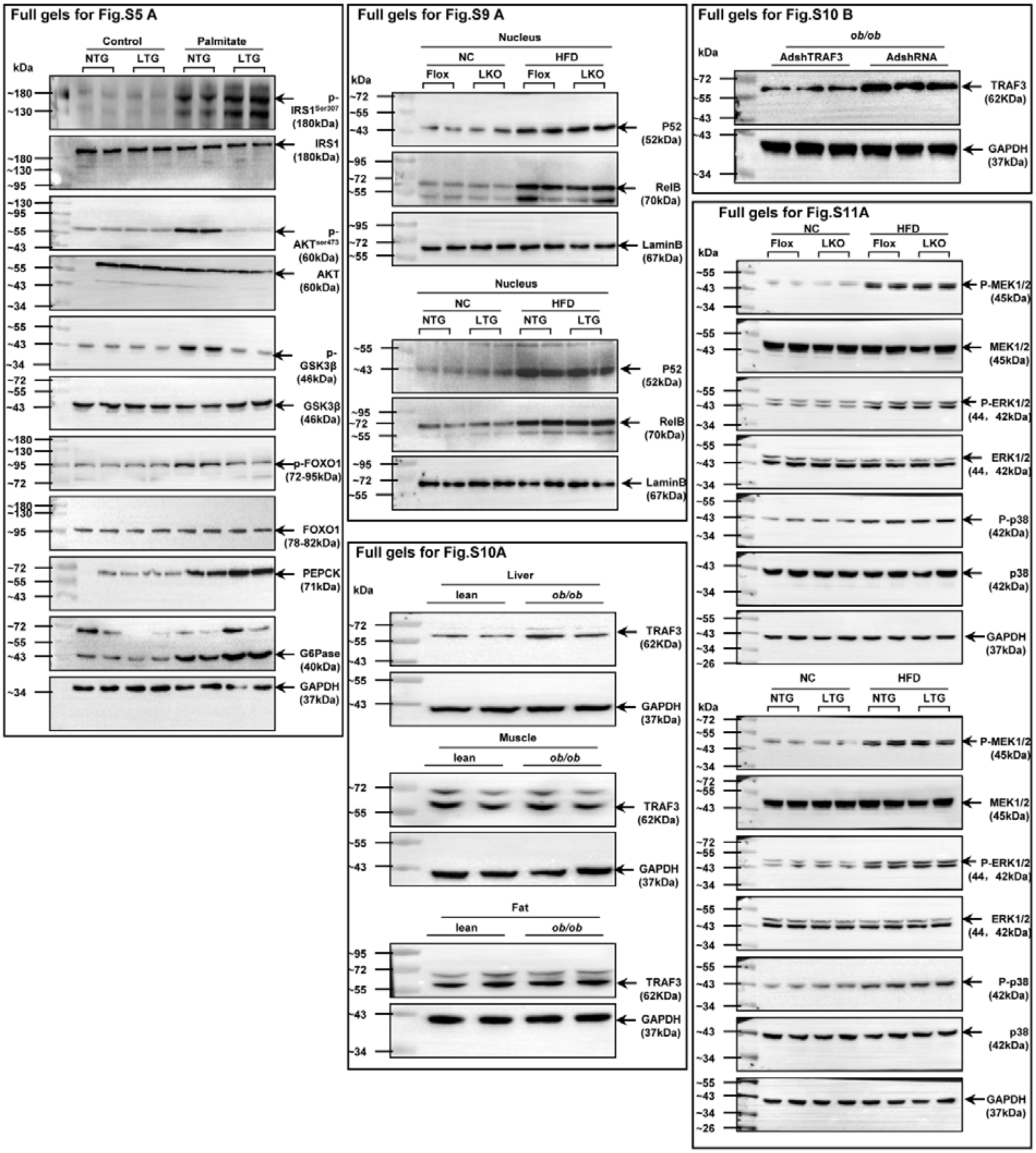

Supplementary Figure 13. Full gel scans relating to indicated figures (continued).

Supplementary Table 1. Serum levels of aminotransferase in mice at 24 week after high-fat diet treatment.

Groups	TRAF3-flox	TRAF3-LKO	NTG	TRAF3-LTG
**Serum aminotransferase**				
ALT (U/l)	88.73 ± 8.74	48.80 ± 2.25 #	88.73 ± 10.02	136.20 ± 5.57 #
AST (U/l)	140.91 ± 16.31	55.70 ± 2.31 #	147.18 ± 10.99	190.10 ± 11.77 #
ALP (U/l)	174.73 ± 15.28	61.30 ± 3.40 #	175.45 ± 11.89	212.60 ± 10.29 #

#*P*<0.05 vs. corresponding TRAF3-flox or NTG group.

Supplementary Table 2. The primers used to genotype the conditional cardiac-specific TRAF3 knockout mice.

Primer name	Primer
P1	TTGCGGAACCCTTCGAAGTTCC
P2	AGGAGGCACAGCTGAAGCTGTG
P3	AGCTTCAGAAGGCCACAGAGTT
P4	CTGGTCTTGGGCTATCTACAAACTC
P5	TGCTTTGGCCAAATTGTTACA
P6	GTACACTGTAGCTATCTTCAGATACACC

P1+P2 to detect the gene targeting, Product: 1740bp
P3+P4 to detect the Flox allele or FloxNeo allele, Product: WT allele→320bp; FloxNeo allele→1.92kb, Flox allele→406bp
P5+P6 to detect the existence of left Loxp site, Product: △LoxP→200bp; LoxP insert→240bp

Supplementary Table 3. The clinical information of NAFLD patients and donors.

Type	Gender	Age (years)	BMI (kg/m^2)	AST (U/L)	ALT (U/L)	Cholesterol (mg/dL)	Triglycerides (mg/dL)	HDL (mg/dL)	LDL (mg/dL)	FBS (mg/dL)	Insulin (μU/mL)
NAFLD	Male	38	31.24	59	77	159	184	45	138	95	12.5
NAFLD	Male	42	30.45	55	62	154	171	42	123	82	11.6
NAFLD	Female	45	35.63	62	91	168	198	42	150	102	13.4
NAFLD	Female	49	30.45	49	65	158	172	44	122	92	12
NAFLD	Female	52	34.61	64	89	172	185	36	144	94	14.1
NAFLD	Male	53	39.18	56	85	170	182	38	146	98	13.4
NAFLD	Male	56	36.88	66	89	188	203	34	157	104	15.2
Normal	Male	32	22.4	20	26	135	140	62	84	76	6.5
Normal	Female	42	24.2	19	28	136	148	56	85	80	7.2
Normal	Male	46	23.53	18	24	130	145	60	82	78	6.8
Normal	Female	51	21	18	25	128	136	68	76	75	6.2

Supplementary Table 4. Histologic Features of NAFLD Patients.

Type	Gender	Age (years)	Steatosis (0-3)	Lobular inflammation (0-3)	Ballooning (0-2)	Fibrosis (0-4)	NAS (1-8)
NAFLD	Male	38	3	2	0	0	5
NAFLD	Male	42	3	1	0	0	4
NAFLD	Female	45	3	2	1	0	6
NAFLD	Female	49	3	2	0	0	5
NAFLD	Female	52	3	2	0	0	5
NAFLD	Male	53	3	2	1	0	6
NAFLD	Male	56	3	3	1	1	7

Supplementary Table 5. Primers for Real-time PCR detection.

Gene		Sequence5'---3'
GAPDH	Forward	ACTCCACTCACGGCAAATTC
	Reverse	TCTCCATGGTGGTGAAGACA
TRAF3-H	Forward	ACTGCAAGAGTCAGGTTCCG
	Reverse	CAAGTGTGCACTCAACTCGC
TRAF3-M	Forward	ACTGAGCTGGAGAGCGTAGA
	Reverse	GCGCTTGTAGTCACGGATCT
PEPCK	forward	TGCCCCAGGCAGTGAGGAAGTT
	reverse	GTCAGTGAGAGCCAGCCAACAGT
G6Pase	forward	TCTGTCCCGGATCTACCTTG
	reverse	GCTGGCAAAGGGTGTAGTGT
ABCG1	forward	TGAACCCGTTTCTTTGGCACCG
	reverse	AGTCCCGCATGATGCTGAGGAA
CYP7A1	forward	TCAAAGAGCGCTGTCTGGGTCA
	reverse	TTTCCCGGGCTTTATGTGCGGT
SREBP-1c	forward	CACTTCTGGAGACATCGCAAAC
	reverse	ATGGTAGACAACAGCCGCATC
ACCα	forward	GGCCAGTGCTATGCTGAGAT
	reverse	AGGGTCAAGTGCTGCTCCA
FAS	forward	CTGCGGAAACTTCAGGAAATG
	reverse	GGTTCGGAATGCTATCCAGG
SCD1	forward	TCTTCCTTATCATTGCCAACACCA
	reverse	GCGTTGAGCACCAGAGTGTATCG
CD36	forward	TGGGTTTTGCACATCAAAGA
	reverse	GATGGACCTGCAAATGTCAGA
FABP1	forward	TGGTCCGCAATGAGTTCACCCT
	reverse	CCAGCTTGACGACTGCCTTGACTT
FATP1	forward	TGCACAGCAGGTACTACCGCAT
	reverse	TGCGCAGTACCACCGTCAAC
PPAR-α	forward	TATTCGGCTGAAGCTGGTGTAC
	reverse	CTGGCATTTGTTCCGGTTCT
ACOX	forward	CGGAAGATACATAAAGGAGACC
	reverse	AAGTAGGACACCATACCACCC
CPT-1α	forward	AGGACCCTGAGGCATCTATT
	reverse	ATGACCTCCTGGCATTCTCC

MCAD	forward	TGGCGTATGGGTGTACAGGG
	reverse	CCAAATACTTCTTTTTTTGTTGATCA
LCAD	forward	GGAGTAAGAACGAACGCCAA
	reverse	GCCACGACGATCACGAGAT
UCP2	forward	GCTGGTGGTGGTCGGAGATA
	reverse	ACTGGCCCAAGGCAGAGTT
PPAR-γ	forward	ATTCTGGCCCACCAACTTCGG
	reverse	TGGAAGCCTGATGCTTTATCCCCA
PDK4	forward	TTCACACCTTCACCACATGC
	reverse	AAAGGGCGGTTTTCTTGATG
G6PC	forward	TCTGTCCCGGATCTACCTTG
	reverse	GCTGGCAAAGGGTGTAGTGT
HMGCR	forward	ATCATGTGCTGCTTCGGCTGCAT
	reverse	AAATTGGACGACCCTCACGGCT
IL-1β	forward	CCGTGGACCTTCCAGGATGA
	reverse	GGGAACGTCACACACCAGCA
IL-6	forward	AGTTGCCTTCTTGGGACTGA
	reverse	TCCACGATTTCCCAGAGAAC
TNF-α	forward	CATCTTCTCAAAATTCGAGTGACAA
	reverse	TGGGAGTAGACAAGGTACAACCC
MCP1	forward	TAAAAACCTGGATCGGAACCAAA
	reverse	GCATTAGCTTCAGATTTACGGGT
iNOS	forward	TGCGCCTTTGCTCATGACATCGA
	reverse	ATGGATGCTGCTGAGGGCTCTGTT
IL-10	forward	CCAAGCCTTATCGGAAATGA
	reverse	TTTTCACAGGGGAGAAATCG

Supplementary Table 6. Primers for the creation of TRAF3/TAK1 construct.

Primer	Sequence (5' to 3')
TRAF3-1F:	CGCGGATCCATGGAGTCGAGTAAAAAGATG
TRAF3-568R:	CCGCTCGAGTCAGGGATCGGGCAGATCC
TRAF3-267F:	CGCGGATCCAGCAACTCGCTCGAAAAGAA
TRAF3-266R:	CCGCTCGAGCCACTCCTTCAGCAGGTTGA
TRAF3-376R:	CCGCTCGAGCCGAGCCACTTGCCCCGCGCT
TAK1-1F:	CGCGGATCCATGTCTACAGCCTCTGCCGC
TAK1-579R:	CCGCTCGAGTCATGAAGTGCCTTGTCGTT
TAK1-301F:	CGCGGATCCCCTTGTCAGTATTCAGATGA
TAK1-481F:	CGCGGATCCCAGCCTCTAGCACCGTGC
TAK1-300R:	CCGCTCGAG ATACTGTAATGGCTCATCTG
TAK1-480R:	CCGCTCGAG TAGTTGGTGATCCAGTGTAA
